# Risk factors for invasive *Klebsiella pneumoniae* liver abscess syndrome: a meta-analysis

**DOI:** 10.3389/fcimb.2026.1749555

**Published:** 2026-01-27

**Authors:** Aimin Chen, Zhilin Luo, Yibin Peng

**Affiliations:** Laboratory Department, The First Hospital of Putian City, Putian, Fujian, China

**Keywords:** diabetes, invasive *Klebsiella pneumoniae* liver abscess, liver disease, meta-analysis, phlebitis, risk factors

## Abstract

**Objectives:**

Invasive *Klebsiella pneumoniae* liver abscess syndrome (IKPLAS) is a life-endangering condition. This meta-analysis sought to identify factors that could potentially serve as risk factors for IKPLAS.

**Methods:**

Eight databases were retrieved. The Newcastle-Ottawa Scale and AHRQ criteria were utilized to assess the studies for quality. Stata software was used for pooling the effect size odds ratio (OR) and for subgroup analysis, sensitivity analysis, and regression analysis. Publication bias was examined leveraging funnel plots and Egger’s test.

**Results:**

A total of 18 studies (cross-sectional, cohort, case-control studies) were included, with 3,133 patients. The meta-analysis revealed that the following factors were linked to a significantly elevated risk of IKPLAS: history of smoking or alcohol consumption (OR = 1.61, 95% CI: 1.05, 2.45, p = 0.028), diabetes (OR = 1.93, 95% CI: 1.49, 2.51, p<0.001), liver disease (OR = 1.66, 95% CI: 1.01, 2.73, p = 0.044), elevated sequential organ failure assessment (SOFA) score (OR = 1.71, 95% CI: 1.27, 2.30, p<0.001), septic shock (OR = 3.30, 95% CI: 1.70, 6.37,p<0.001), hepatic abscess in the left lobe (OR = 1.51, 95% CI: 1.02, 2.24, p = 0.039), phlebitis (OR = 21.01, 95% CI: 10.24, 43.11, p<0.001), procalcitonin (PCT) (OR = 1.01, 95% CI: 1.01, 1.02, p<0.001), fasting blood glucose (FBG) (OR = 1.21, 95% CI: 1.11, 1.32, p<0.001), prothrombin time (PT) (OR = 1.15, 95% CI: 1.01, 1.30, p = 0.032), total bilirubin (OR = 1.02, 95% CI: 1.01, 1.02, p<0.001), hypervirulent phenotype (OR = 2.17, 95% CI: 1.15, 4.10, p = 0.017), K1 serotype (OR = 4.79, 95% CI: 1.79, 12.76, p = 0.002). No other risk factors were found.

**Conclusion:**

This study identified multiple risk factors significantly associated with IKPLAS, providing evidence for risk assessment and prevention strategies.

## 1 Introduction

In Asia, it has been confirmed that *Klebsiella pneumoniae* (KP) has replaced other pathogens as the primary pathogenic bacterium of community-acquired pyogenic liver abscess. Particularly in Taiwan, mainland China, South Korea, and India, the detection rates of KP soar to between 70% and 90% (Liu et al., 1986; Yin et al., 2021; Behera et al., 2025; Dhamelia et al., 2025; Juan et al., 2025; Si et al., 2025). The elevated incidence is tightly linked to the pervasive presence of K1/K2 serotypes in hypervirulent KP (hvKP) strains and their associated virulence genes (Ko et al., 2002; Chung et al., 2007). Serotypes K1 and K2 are predominant. A study conducted in Shandong, China has found that the detection rates for K1 and K2 in hvKP strains are 25.00% and 36.67%, respectively, which are significantly higher than the detection rates in classical KP strains, at 12.00% and 9.20% (Han et al., 2025). These patterns are also noted in other Asian countries such as Iran and Malaysia, where K1 and K2 serotypes frequently coexist with hvKP virulence genes, thereby amplifying their pathogenic potential (Han et al., 2025; Khairuddin et al., 2025; Loiodice et al., 2025; Zhou et al., 2025). These hypervirulent strains harbor multiple virulence genes, including rmpA, rmpA2, iucA, and iroB, which are intricately connected to their ability to adhere, mechanisms of iron acquisition, and capsule formation (Behera et al., 2025; Han et al., 2025; Loiodice et al., 2025; Wu et al., 2025). Specifically, the sequence type (ST) 23 of hvKP characteristically harbors virulence factors such as iucABCD, iroEN, and rmpA, facilitating the development of liver abscesses via advanced mechanisms of immune evasion and spread of the bacteria (Dhamelia et al., 2025; Khairuddin et al., 2025; Loiodice et al., 2025; Xin et al., 2025). It is important to note that hvKP strains have disseminated from Asia to Europe and the Americas, progressively emerging as a worldwide public health concern (Ko et al., 2002; Pope et al., 2011; Ejikeme et al., 2021). KP liver abscess (KPLA) represents a severe infection with a mortality rate of 3%–17% among hospitalized patients. If it progresses to invasive *Klebsiella pneumoniae* liver abscess syndrome (IKPLAS), the mortality rate surges to 20%–35%. IKPLAS is characterized by concurrent distant metastatic infections in the eyes, lungs, and central nervous system (CNS). This is accompanied by significantly increased intensive care unit admission rates and permanent visual or neurological disabilities, imposing a substantial burden. Therefore, IKPLAS has become an acute infection requiring urgent intervention (Zhang et al., 2022; Chang et al., 2023).

Early identification of high-risk patients is crucial for preventing IKPLAS. However, existing literature presents inconsistent and even contradictory conclusions regarding predictive risk factors for IKPLAS. This represents a bottleneck in clinical practice. A retrospective study in South Korea (Shin et al., 2013) has proposed that liver abscess size ≤ 5.8 cm and altered state of consciousness at admission are independent predictors of metastatic infection (Shin et al., 2013). A Chinese study focusing on imaging characteristics (Chang et al., 2015) has suggested that unilocular abscess and smaller abscess diameters are significantly associated with concurrent septic pulmonary embolism (Chang et al., 2015). Additionally, indicators, including diabetes, male gender, decreased platelet count, and sequential organ failure assessment (SOFA) score ≥ 4, have been individually validated by different cohorts. However, effect sizes vary significantly and are even contradictory (Li et al., 2020; Li et al., 2023; Gu et al., 2025; Sun et al., 2025). The fragmented evidence makes it difficult to establish unified, operable high-risk screening thresholds and early warning models for IKPLAS in clinical practice (Yoon et al., 2014; Wang et al., 2022).

To date, there have been no systematic reviews and meta-analyses to quantitatively integrate risk factors for IKPLAS. This study synthesized fragmented evidence to determine risk factors for IKPLAS. It aimed to provide scientific, precise, evidence-based medical support to reduce the burden of this fatal and disabling infection.

## 2 Materials and methods

This study was conducted in conformity with the Preferred Reporting Items for Systematic Reviews and Meta-Analyses (PRISMA) guidelines (Page et al., 2021). The study protocol has been registered in the international prospective systematic review registry PROSPERO (CRD420251143764).

### 2.1 Literature search

PubMed, Embase, Cochrane Library, Web of Science, CNKI, WanFang, VIP, and Sinomed databases were retrieved to gather publications dated as of September 4, 2025. Subject headings and free-text terms were used in combination as search terms. The medical subject headings used were “Klebsiella pneumoniae” and “liver abscess”. The synonyms search employed terms such as “bacillus pneumoniae” and “abscess of the liver” alongside various others. Additionally, references from relevant articles and grey literature were manually searched to acquire eligible studies. The search strategy is depicted in **Supplementary Table S1**.

### 2.2 Inclusion and exclusion criteria

Studies meeting the following criteria were included: (1) Subjects: Patients with liver abscess suggested by imaging and KP strains isolated from liver biopsy or blood culture. (2) Exposure group: Patients with KP-induced liver abscess complicated by metastatic infections (e.g., lung abscess, endophthalmitis, meningitis, necrotizing fasciitis). Control group: Patients with KP-induced liver abscess without metastatic infections. (3) Study type: observational studies. (4) Outcome measures: Risk factors for IKPLAS.

The following studies were excluded: (1) Animal or cell studies, case reports, experiment protocols, reviews, letters, editorials, conference papers; (2) Publications with missing data or critical mistakes (errors in the confidence intervals of outcome data); (3) Duplicate publications; (4) Publications with full text unavailable; (5) Studies enrolling overlapping subjects.

### 2.3 Data extraction

Retrieved articles were imported into EndNote. Based on the above criteria, two researchers (Aimin Chen and Yibin Peng) screened the titles and abstracts independently, followed by a full-text review. Dissents were resolved through discussion or referred to a third researcher (Zhilin Luo). Data were extracted independently by two researchers with a pre-designed spreadsheet, capturing information covering first author, publication year, country/region, basic information of study subjects, and outcome measures.

### 2.4 Quality assessment

Two authors (Aimin Chen and Yibin Peng) appraised the quality of eligible studies independently using the Newcastle-Ottawa Scale (NOS) (Stang, 2010). This 8-item scale covers three dimensions: selection, comparability, and exposure. Each study was assigned an NOS score of 0 to 9, with studies scoring 6 or higher classified as high-quality studies and those scoring 5 or lower as low-quality studies.

For quality assessment of cross-sectional studies, an 11-item checklist, as recommended by the Agency for Healthcare Research and Quality (AHRQ), was employed (Rostom A et al., 2004). Studies scoring 0–3 were rated low-quality; studies scoring 4–7 were rated medium-quality; and studies scoring 8–11 were rated high-quality.

### 2.5 Statistical analysis

Statistical analysis was implemented utilizing STATA 18. Risk factors for IKPLAS were assessed by calculating OR and 95% confidence interval (CI). Heterogeneity was evaluated using Cochrane I² statistics. P < 0.10 and I² > 50% implied statistical heterogeneity. A random-effects model (DerSimonian-Laird model) (Veroniki et al., 2016) was utilized when heterogeneity was significant; otherwise, a fixed-effects model (inverse variance model) was utilized. To assess the stability of results, a sensitivity analysis was implemented. Subgroup analyses by confounder control and study design and meta-regression (residual maximum likelihood) were implemented to ascertain sources of heterogeneity. Funnel plots were utilized to assess publication bias for outcomes disclosed in more than 10 studies. Publication bias was quantified utilizing Egger’s test, with P < 0.05 signifying significant bias (Sterne et al., 2011).

## 3 Results

### 3.1 Literature search and screening process

**Figure 1:**
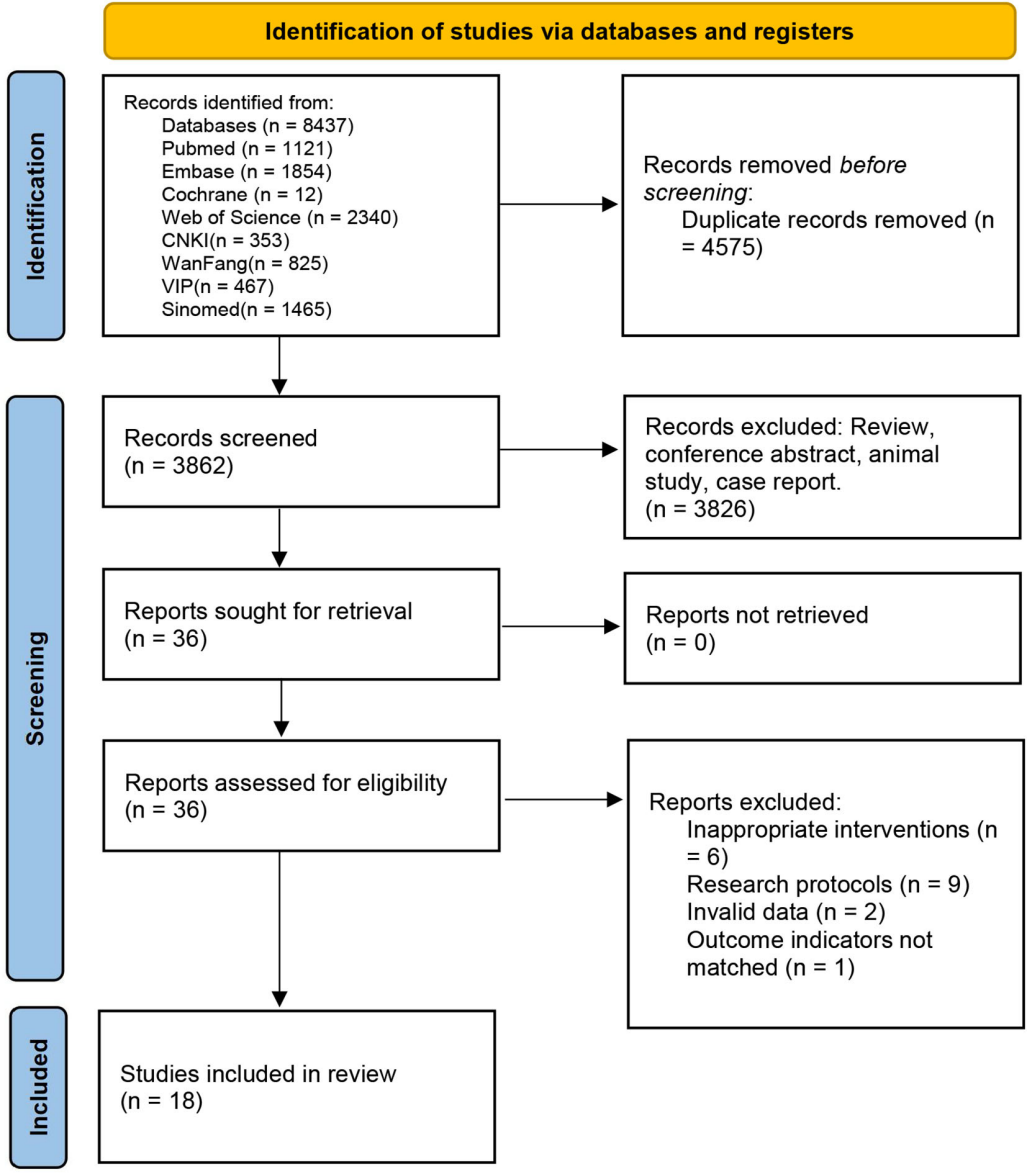
Literature search and screening flowchart.

A total of 8,437 articles were searched, and 4,575 articles remained after eliminating duplicates. After initial screening by titles and abstracts, 3,826 articles were eliminated. The remaining articles were fully reviewed and strictly screened according to the inclusion and exclusion criteria. Finally, 18 studies were incorporated. The screening process is depicted in **Figure 1**.

### 3.2 Basic characteristics of incorporated studies and quality evaluation

The 18 incorporated studies (Lee et al., 2010; Shin et al., 2013; Yoon et al., 2014; Lee et al., 2018; Li et al., 2020; Wang et al., 2022; Zhang et al., 2022; Li et al., 2023; Feng et al., 2024; Hu et al., 2024; Wu et al., 2024; Zhang et al., 2024; Zhao et al., 2024; Zheng et al., 2024; Gu et al., 2025; Lv et al., 2025; Sun et al., 2025; Zhu et al., 2025) involved a total of 3,133 patients from two countries, China and South Korea. Among them, 61% were male and 39% were female. The ages of the patients varied from 52 to 64 years. Basic characteristics of the incorporated studies are listed in **Table 1**. The incorporated studies consistently defined IKPLAS as a severe infectious disease caused by *Klebsiella pneumoniae* primarily characterized by pyogenic lesions in the liver, accompanied by disseminated infections in multiple sites throughout the body (**Supplementary Table S2**).

**Table 1:** Basic characteristics of the included studies.

First author	Year	Study design	Country	Sample size	Age	Gender (Male/Female)	Patient type	Outcome indicator
L. Gu	2025	cross-sectional	China	208	52.25 (0.92)	174/34	unlimited	1, 2, 4, 11, 13, 14, 15, 16, 17, 18, 21, 28, 29, 33, 34, 35, 40, 49, 50
F. Li	2023	Cohort	China	51	54.82(13.63)	28/23	unlimited	1, 3, 4, 11, 28, 29, 30, 31, 32, 35, 36, 37, 38, 39, 52
H. Sun	2025	Cohort	China	291	63.95(3.85)	215/76	unlimited	1, 2, 4, 5, 6, 7, 8, 9, 10, 12, 13, 22, 26, 40, 46, 48, 53
L. Li	2020	Cohort	China	81	59(17.03)	57/24	unlimited	1, 2, 3, 4, 5, 7, 8, 9, 10, 14, 19, 21, 23, 24, 25,
S. U. Shin	2013	Cohort	Korea	60	/	43/17	unlimited	27, 28, 29, 30, 32, 35, 36
H. Wang	2022	Cohort	China	382	58.96(2.72)	223/159	unlimited	1, 2, 4, 10, 11, 26, 28, 30, 34, 37, 39, 41, 42,
C. Y. Feng	2024	case-control	China	213	61.4(12.0)	60/153	combined diabetes	1, 5, 7, 11, 13, 14, 16, 19, 27, 29, 30, 33, 34, 35, 41, 42,
C.-H. Lee	2010	cross-sectional	China	91	/	58/33	unlimited	1, 2, 4, 5, 6, 10, 11, 19, 22, 23, 24, 25, 26,
J. Zhu	2025	case-control	China	68	/	/	unlimited	1, 2, 3, 4, 5, 6, 7, 11, 27, 40, 42, 43, 44, 45, 50, 51, 53, 54, 55
J. H. Yoon	2014	Cohort	Korea	161	/	100/61	unlimited	1, 2, 3, 4, 5, 10, 11, 20, 21, 46, 47, 54
Z. Zhang	2022	cross-sectional	China	16	/	10/6	unlimited	1, 4, 10, 11, 22, 23, 24, 28, 30, 37, 39,
C. H. Lee	2018	cross-sectional	China	175	/	64/111	combined diabetes	1, 2, 5, 6, 7, 8, 10, 11, 19, 23, 42,
S.Wu	2024	Cohort	China	68	56.5(18.3)	36/32	unlimited	1, 4, 14, 15, 28, 34, 35, 37, 38, 39,
Q.Hu	2024	case-control	China	201	56.0(12.3)	131/70	unlimited	1, 4, 11, 13, 14, 15, 16, 17, 18, 19, 43, 46, 53, 54, 55
F.F.Zheng	2024	Cohort	China	75	57.2(11.5)	49/26	unlimited	1, 3, 4, 5, 10, 11, 14, 15, 16, 17, 18, 19, 20, 21, 27, 29, 30, 33, 34, 35, 43,
P.C.Zhao	2024	Cohort	China	161	/	103/58	unlimited	1, 3, 4, 5, 10, 19, 30,
X.F.Lv	2025	Cohort	China	389	59.04(2.38)	244/145	unlimited	1, 2, 4, 7, 11, 13, 27, 28, 31, 40, 41, 43, 44, 45, 46, 47, 48, 49, 50, 51, 52, 53, 54, 55
T.Z.Zhang	2024	Cohort	China	442	/	271/171	unlimited	1, 3, 4, 5, 8, 9, 10, 11, 12, 13, 14, 16, 17, 18, 21, 29, 30, 33, 34, 35, 43, 55

1 Gender (Male), 2 Age, 3 Hypertension, 4 Diabetes mellitus, 5 Liver disease, 6 Chronic kidney disease, 7 Cardiovascular disease, 8 Chronic pulmonary disease, 9 Neurologic disease, 10 Tumor and autoimmune, 11 Biliary diseases, 12 Septic shock, 13 Sequential organ failure assessment (SOFA), 14 Abdominal symptoms, 15 Fever, 16 Poor appetite, 17 Weakness, 18 Chills/shivers, 19 Smoking or drinking history, 20 Invasive procedure, 21 Abdominal surgical history, 22 Hypervirulent phenotype, 23 K1 serotype, 24 K2 serotype, 25 Vigilance gene positive, 26 Drug-resistant strains, 27 Abscess size, 28 Gas in abscess, 29 Single, 30 Multiple, 31 No. of abscesses, 32 Simple leaf, 33 Left lobe, 34 Right lobe, 35 Both lobes, 36 Texture, 37 Septa, 38 Abnormal perfusion, 39 Phlebitis, 40 Procalcitonin (PCT), 41 Fasting blood glucose (FBG), 42 Glycated hemoglobin, 43 Hemoglobin, 44 Prothrombin time (PT), 45 Activated coagulation time of whole blood (APTT), 46 Platelet (PLT), 47 Albumin, 48 Creatinine (Cr), 49 D-dimer, 50 Alanine aminotransferase (ALT), 51 Aspartate aminotransferase (AST), 52 Total bilirubin, 53 White blood cell (WBC), 54 C-reactive protein (CRP), 55 Neutrophile granulocyte.

Cohort studies: Most included studies scored above 6 points, signifying high quality. One study was assigned a score of 5 points. This was because the subjects were already ill at baseline, the exposed and unexposed groups were not adjusted for confounders, and follow-up for the studied disease was not complete.

Case-control studies: All included studies scored above 6 points, signifying high quality.

Cross-sectional studies: Among the 4 included studies, 1 scored 8 points, signifying high quality. Two studies scored 7 points, primarily due to the absence of explanation for the exclusion of any patients from the analysis and failure to adjust the exposed and unexposed cohorts for confounders. One study scored 6 points, mainly due to unclear data sources and failure to adjust for confounders.

### 3.3 Results of meta-analysis

#### 3.3.1 Pooled results of each outcome measure

##### 3.3.1.1 Basic characteristics and medical history

The pooled results indicated that, among patients with KPLA, the risk of invasiveness in individuals with a history of smoking or alcohol consumption was significantly higher compared to the control group (OR = 1.61, 95% CI: 1.05, 2.45, p = 0.028). The risk of invasiveness in the diabetes group was significantly higher as compared to the control group (OR = 1.93, 95% CI: 1.49, 2.51, p<0.001). The risk of invasiveness in the liver disease group was significantly higher compared to the control group (OR = 1.66, 95% CI: 1.01, 2.73, p = 0.044). The risk of invasiveness in the septic shock group was significantly higher compared to the control group (OR = 3.30, 95% CI: 1.70, 6.37, p<0.001). The risk of invasiveness in individuals with elevated SOFA was significantly higher compared to the control group (OR = 1.71, 95% CI: 1.27, 2.30, p<0.001). Results without statistical significance are presented in **Table 2**.

**Table 2:** Basic characteristics and medical history.

Influencing factor	Number of included studies	Heterogeneity analysis		Model	Meta-analysis results	
		*I^2^(%)*	P value		OR (95%CI)	P value
Gender (Male)	17	31.3	0.106	fixed	1.11(0.90, 1.37)	0.332
Age	8	24.1	0.237	fixed	0.99(0.98, 1.01)	0.216
Fever	4	0.8	0.388	fixed	2.00(0.79, 5.03)	0.142
Poor appetite	5	22.7	0.270	fixed	1.01(0.66, 1.55)	0.948
Weakness	4	0.0	0.592	fixed	0.85(0.54, 1.33)	0.480
Chills/shivers	4	27.3	0.248	fixed	1.15(0.75, 1.77)	0.516
Smoking or drinking history	7	0.0	0.896	fixed	**1.61(1.05, 2.45)**	**0.028**
Hypertension	7	54.2	0.041	random	0.78(0.38, 1.60)	0.500
Diabetes mellitus	15	45.0	0.030	fixed	**1.93(1.49, 2.51)**	<0.001
Liver disease	10	0.0	0.950	fixed	**1.66(1.01, 2.73)**	**0.044**
Chronic kidney disease	4	0.0	0.581	fixed	1.61(0.96, 2.70)	0.069
Cardiovascular disease	6	47.4	0.091	fixed	1.26(0.84, 1.90)	0.271
Chronic pulmonary disease	4	25.2	0.261	fixed	0.84(0.38, 1.82)	0.653
Neurologic disease	3	27.1	0.253	fixed	0.95(0.49, 1.86)	0.892
Tumor and autoimmune disease	10	0.0	0.756	fixed	0.82(0.51, 1.30)	0.396
Biliary diseases	13	2.3	0.423	fixed	0.91(0.67, 1.25)	0.569
Septic shock	2	0.0	0.843	fixed	**3.30(1.70, 6.37)**	<0.001
SOFA	6	88.5	<0.001	random	**1.71(1.27, 2.30)**	<0.001
Abdominal symptoms	6	35.2	0.160	fixed	**0.52(0.36, 0.76)**	**0.001**
Invasive procedure	2	0.0	0.870	fixed	1.28(0.36, 4.58)	0.709
Abdominal surgical history	5	0.0	0.653	fixed	0.72(0.29, 1.82)	0.487

Bold values are highlighted for visual emphasis of key results.

Red-highlighted items indicate critical revisions made per standard practice.

##### 3.3.1.2 Imaging indicators

Pooled results showed that among patients with KPLA, the phlebitis group exhibited a substantially higher risk of invasiveness as compared to the control group (OR = 21.01, 95% CI: 10.24, 43.11, p<0.001). The left hepatic lobe abscess group exhibited a substantially higher risk of invasiveness as compared to the control group (OR = 1.51, 95% CI: 1.02, 2.24, p = 0.039). Results without statistical significance are listed in **Table 3**.

**Table 3:** Imaging parameters.

Influencing factor	Number of included studies	Heterogeneity analysis		Model	Meta-analysis results	
		*I^2^(%)*	P value		OR (95%CI)	P value
Abscess size	5	74.2	0.004	random	0.83(0.67, 1.02)	0.081
Gas in abscess	7	9.4	0.357	fixed	1.51(0.96, 2.37)	0.072
Single	6	0.0	0.680	fixed	0.90(0.59, 1.38)	0.633
Multiple	8	29.5	0.193	fixed	1.09(0.78, 1.51)	0.613
No. of abscesses	2	0.0	0.516	fixed	1.47(0.81, 2.68)	0.205
Simple leaf	2	84.0	0.012	random	0.95(0.01, 91.84)	0.984
Left lobe	4	0.0	0.401	fixed	**1.51(1.02, 2.24)**	**0.039**
Right lobe	6	0.0	0.863	fixed	1.02(0.68, 1.54)	0.912
Both lobes	7	31.8	0.186	fixed	0.92(0.48, 1.77)	0.802
Texture	2	55.0	0.136	fixed	0.76(0.27, 2.12)	0.606
Septa	4	71.2	0.015	random	1.02(0.25, 4.14)	0.974
Abnormal perfusion	2	73.8	0.051	random	0.22(0.04, 1.11)	0.067
Phlebitis	4	0.0	0.857	fixed	**21.01(10.24, 43.11)**	<0.001

Bold values are highlighted for visual emphasis of key results.

Red-highlighted items indicate critical revisions made per standard practice.

###### 3.3.1.2.1 Subgroup analysis

Subgroup analyses by confounder control and study design were performed (**Table 4**). In the subgroup analysis of the gas-containing liver abscess group, the risk of invasiveness in the cohort studies was significantly higher than in other groups (OR = 1.72, CI: 1.06, 2.80, p=0.029).

**Table 4:** Subgroup analyses.

Influencing factor	Number of included studies	Heterogeneity analysis		Model	Meta-analysis results	
		*I^2^(%)*	P value		OR (95%CI)	P value
Gender (Male)						
cross-sectional	4	53.4	0.092	fixed	0.78(0.49, 1.26)	0.315
cohort	10	15.8	0.297	fixed	1.30(1.00, 1.68)	0.050
case-control	3	5.6	0.347	fixed	0.90(0.53, 1.52)	0.687
		31.3	0.106	fixed	1.11(0.90, 1.37)	0.332
Age						
adjusted	2	84.4	0.011	fixed	1.02(0.96, 1.07)	0.580
Not adjusted	6	0.0	0.845	fixed	0.99(0.98, 1.00)	0.155
		24.1	0.237	fixed	0.99(0.98, 1.01)	0.216
Smoking or drinking history						
cross-sectional	2	0.0	0.954	fixed	1.64(0.54, 4.93)	0.379
cohort	2	0.0	0.677	fixed	1.14(0.55, 2.34)	0.722
case-control	3	0.0	0.866	fixed	2.01(1.11, 3.63)	0.021
		0.0	0.896	fixed	1.61(1.05, 2.45)	0.028
Diabetes mellitus						
adjusted	8	67.5	0.003	fixed	1.72(1.05, 2.82)	0.032
Not adjusted	7	0.0	0.732	fixed	2.02(1.49, 2.75)	<0.001
cross-sectional	3	0.0	0.602	fixed	1.25(0.61, 2.59)	0.541
cohort	10	59.6	0.008	fixed	1.98(1.46, 2.68)	<0.001
case-control	2	0.0	0.789	fixed	2.62(1.28, 5.36)	0.009
		45.0	0.030	fixed	1.93(1.49, 2.51)	<0.001
Hepatopathy						
cross-sectional	2	0.0	0.988	fixed	2.02(0.65, 6.30)	0.225
cohort	6	0.0	0.908	fixed	1.61(0.85, 3.06)	0.146
case-control	2	39.1	0.200	fixed	1.53(0.53, 4.44)	0.432
		0.0	0.950	fixed	1.66(1.01, 2.73)	0.044
Tumor and autoimmune disease						
cross-sectional	3	0.0	0.596	fixed	0.50(0.17, 1.41)	0.190
cohort	7	0.0	0.715	fixed	0.92(0.55, 1.55)	0.765
		0.0	0.756	fixed	0.82(0.51, 1.30)	0.396
Biliary diseases						
cross-sectional	4	0.0	0.741	fixed	0.65(0.30, 1.42)	0.280
cohort	6	35.1	0.173	fixed	0.88(0.57, 1.37)	0.579
case-control	3	0.0	0.376	fixed	1.14(0.60, 1.99)	0.642
		2.3	0.423	fixed	0.91(0.67, 1.25)	0.569
Abscess size						
cohort	3	72.2	0.028	random	0.71(0.49, 1.03)	0.072
case-control	2	85.7	0.008	random	0.93(0.63, 1.38)	0.724
		74.2	0.004	random	0.83(0.67, 1.02)	0.081
Gas in abscess						
adjusted	2	0.0	0.863	fixed	3.17(0.84, 12.01)	0.090
Not adjusted	5	23.9	0.262	fixed	1.37(0.85, 2.22)	0.193
cross-sectional	2	0.0	0.377	fixed	0.72(0.22, 2.30)	0.577
cohort	5	0.0	0.406	fixed	1.72(1.06, 2.80)	0.029
		9.4	0.357	fixed	1.51(0.96, 2.37)	0.072
Thrombophlebitis						
adjusted	2	0.0	0.794	fixed	22.53(8.99, 56.48)	<0.001
Not adjusted	2	0.0	0.423	fixed	18.82(5.94, 59.65)	<0.001
		0.0	0.857	fixed	21.01(10.24, 43.11)	<0.001
Glycated hemoglobin						
adjusted	2	0.0	0.603	random	1.61(1.17, 2.20)	0.434
Not adjusted	2	90.1	0.001	random	1.94(0.37, 10.29)	0.003
		86.9	<0.001	random	1.60(0.92, 2.78)	0.094
Hemoglobin						
adjusted	2	0.0	0.946	fixed	0.97(0.94, 1.000)	0.026
Not adjusted	3	0.0	0.777	fixed	0.99(0.98, 1.01)	0.333
cohort	3	26.4	0.257	fixed	0.99(0.98, 1.00)	0.098
case-control	2	0.0	0.525	fixed	0.99(0.97, 1.02)	0.458
		0.0	0.537	fixed	0.99(0.98, 1.007)	0.070
WBC						
cohort	2	44.3	0.180	fixed	1.01(0.98, 1.04)	0.358
case-control	2	0.0	0.607	fixed	0.98(0.86, 1.10)	0.706
		0	0.495	fixed	1.01(0.98, 1.04)	0.419
CRP						
cohort	2	0.0	0.579	fixed	1.01(1.01, 1.01)	<0.001
case-control	2	0.0	0.837	fixed	1.01(1.00, 1.01)	0.248
		0.0	0.785	fixed	1.01(1.00, 1.01)	<0.001
Neutrophile granulocyte						
adjusted	2	92.7	<0.001	random	1.01(0.88, 1.16)	0.835
Not adjusted	2	0.0	0.968	random	1.00(0.96, 1.03)	0.914
cohort	2	92.7	<0.001	random	1.01(0.88, 1.16)	0.835
case-control	2	0.0	0.968	random	1.00(0.96, 1.03)	0.914
		78.3	0.003	random	1.01(0.94, 1.08)	0.821

Red-highlighted items indicate critical revisions made per standard practice.

##### 3.3.1.3 Laboratory indicators

Pooled results showed that among patients with KPLA, the high procalcitonin (PCT) group exhibited a substantially higher risk of invasiveness than the control group (OR = 1.01, 95% CI: 1.01, 1.02, p<0.001). The high fasting blood glucose (FBG) group exhibited a substantially higher risk of invasiveness than the control group (OR = 1.21, 95% CI: 1.11, 1.32, p<0.001). The high prothrombin time (PT) group exhibited a substantially higher risk of invasiveness than the control group (OR = 1.15, 95% CI: 1.01, 1.30, p = 0.032). The high total bilirubin group exhibited a substantially higher risk of invasiveness than the control group (OR = 1.02, 95% CI: 1.01, 1.02, p<0.001). Results without statistical significance are presented in **Table 5**.

**Table 5:** Laboratory parameters.

Influencing factor	Number of included studies	Heterogeneity analysis		Model	Meta-analysis results	
		*I^2^(%)*	P value		OR (95%CI)	P value
PCT	4	15.2	0.316	fixed	**1.01(1.01, 1.02)**	<0.001
FBG	3	46.9	0.152	fixed	**1.21(1.11, 1.32)**	<0.001
Glycated hemoglobin	4	86.9	<0.001	random	1.60(0.92, 2.78)	0.094
Hemoglobin	5	0.0	0.537	fixed	0.99(0.98, 1.00)	0.070
PT	2	0.7	0.316	fixed	**1.15(1.01, 1.30)**	**0.032**
APTT	2	0.0	0.936	fixed	1.01(0.07, 1.05)	0.721
PLT	4	88.9	<0.001	random	0.99(0.98, 1.01)	0.391
Albumin	2	36.8	0.208	fixed	**0.90(0.84, 0.96)**	**0.003**
Cr	2	1.0	<0.001	fixed	1.01(1.00, 1.01)	0.009
D-dimer	2	0.0	<0.001	fixed	0.98(0.87, 1.10)	0.736
ALT	3	28.3	0.237	fixed	0.99(0.98, 1.00)	0.243
AST	2	0.0	<0.001	fixed	1.00(0.98, 1.02)	0.862
Total bilirubin	2	60.2	0.113	fixed	**1.02(1.01, 1.02)**	<0.001
WBC	4	0.0	0.495	fixed	1.01(0.98, 1.04)	0.419
CRP	4	0.0	0.785	fixed	1.01(1.00, 1.01)	<0.001
Neutrophile granulocyte	4	78.3	0.003	random	1.01(0.94, 1.08)	0.821

Bold values are highlighted for visual emphasis of key results.

Red-highlighted items indicate critical revisions made per standard practice.

###### 3.3.1.3.1 Subgroup analysis

Subgroup analyses were conducted based on confounding factor control and study design (**Table 4**). In the subgroup analysis of the glycated hemoglobin group, the invasive risk was significantly higher in the confounding factor-controlled subgroup compared to other groups (OR = 1.61, CI: 1.17, 2.20, p=0.434).

##### 3.3.1.4 Characteristics of *Klebsiella pneumoniae* isolates

Pooled results showed that among patients with KPLA, the hypervirulent phenotype group exhibited a substantially higher risk of invasiveness than the control group (OR = 2.17, 95% CI: 1.15, 4.10, p = 0.017). The K1 serotype group exhibited a substantially higher risk of invasiveness compared to the control group (OR = 4.79, 95% CI: 1.79, 12.76, p = 0.002). Results without statistical significance are presented in **Table 6**.

**Table 6:** Characteristics of *Klebsiella pneumoniae* isolates.

Influencing factor	Number of included studies	Heterogeneity analysis		Model	Meta-analysis results	
		*I^2^(%)*	P value		OR (95%CI)	P value
Hypervirulent phenotype	3	0.0	0.795	fixed	**2.17(1.15, 4.10)**	**0.017**
K1 serotype	4	37.4	0.187	fixed	**4.79(1.79, 12.76)**	**0.002**
K2 serotype	3	0.0	0.584	fixed	1.20(0.51, 2.85)	0.677
Vigilance gene positive	2	0.0	0.569	fixed	2.20(0.90, 5.40)	0.084
Drug-resistant strains	3	53.2	0.118	fixed	0.97(0.57, 1.65)	0.903

Bold values are highlighted for visual emphasis of key results.

Red-highlighted items indicate critical revisions made per standard practice.

#### 3.3.2 Sensitivity analyses

Sensitivity analyses were implemented to appraise the robustness of results. The studies were sequentially excluded one by one, and changes in effect sizes were evaluated. The analyses demonstrated that most results remained generally stable. However, the results were unstable for liver abscess size. Consequently, the study by P.C. Zhao (Zhao et al., 2024) was excluded, and a reanalysis was performed. After reanalysis, the results were stable. Detailed results of sensitivity analyses are summarized in **Supplementary Figure S1**.

#### 3.3.3 Publication bias

**Figure 2:**
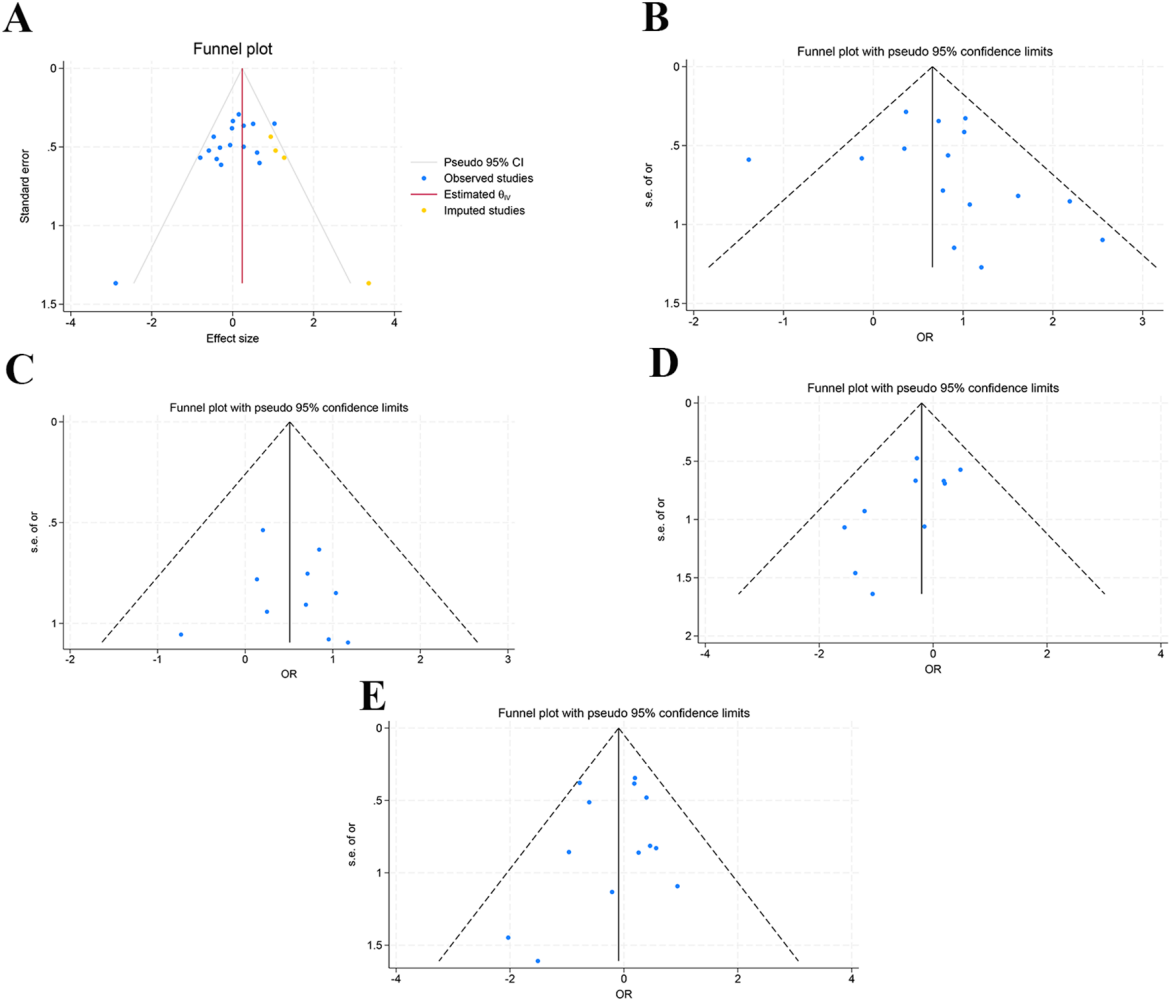
Funnel plots. **(A)** Gender (Male); **(B)** Diabetes; **(C)** Liver disease; **(D)** Tumor and autoimmune diseases; **(E)** Biliary disease.

To assess publication bias in the meta-analysis, funnel plots and Egger’s test were applied for outcomes reported in more than 10 studies. The funnel plot for the male gender was asymmetric. Egger’s test (p = 0.021) suggested potential publication bias. The stability of gender outcomes was assessed using the trim-and-fill method. The results after trim and fill (OR = 1.266, 95% CI: 1.041, 1.540) differed significantly from those before trim and fill (OR = 1.109, 95% CI: 0.900, 1.368), suggesting that publication bias may have affected the stability of gender outcomes. Funnel plots for diabetes (p = 0.392), liver disease (p = 0.777), tumors and autoimmune diseases (p = 0.059), and biliary tract disease (p = 0.649) were symmetrical. Egger’s test denoted no publication bias. Details are illustrated in **Figure 2**.

#### 3.3.4 Meta-regression

For outcomes reported by > 10 studies with significant heterogeneity (publication year, age, sample size, gender), meta-regression was conducted. Potential sources of heterogeneity were explored. Results showed no statistically significant regression results for gender, diabetes, liver disease, tumors, autoimmune diseases, and biliary tract disease (p > 0.05).

## 4 Discussion

This meta-analysis identified major risk factors for IKPLAS. The results revealed significant associations of the following factors with increased risk of IKPLAS: history of smoking or alcohol consumption, diabetes, liver disease, elevated SOFA score, septic shock, hepatic abscess of the left lobe, phlebitis, high PCT, high FBG, high PT, high total bilirubin, hypervirulent phenotype, and K1 serotype. No significant associations were found for other factors.

Diabetes and elevated FBG are recognized risk factors for IKPLAS, consistent with the study by Shin et al. in South Korea. In their study, diabetes patients accounted for 37.1% of all subjects, and the mean FBG at admission for all IKPLAS patients was 9.3 mmol/L, significantly higher than that in the non-metastasis group (6.1 mmol/L) (Wang et al., 2022). Diabetes, particularly with poor glycemic control, acts as a pivotal trigger that shifts Klebsiella pneumoniae from colonization to distant spread, resulting in invasive liver abscess. Chronic hyperglycemia remodels host-pathogen interactions through multiple mechanisms. First, advanced glycation end-products (AGEs) bind to their receptors (RAGE), activating NF-κB/NLRP3 inflammasomes and triggering cytokine storms. This causes damage to the hepatic sinusoidal endothelial barrier, opening a gateway for bacteria to enter the bloodstream. Meanwhile, the hyperosmolar microenvironment suppresses the phagocytic activity of Kupffer cells while diminishing the reactive oxygen species (ROS) burst in neutrophils and the formation of extracellular trap (NET), thus enabling bacteria to evade immune clearance. Furthermore, excessive glucose directly promotes the synthesis of capsular polysaccharide in K1-type hypervirulent strains via the phosphotransferase system (PTS), thus enhancing mucosal barriers and anti-phagocytic capabilities. Finally, the downregulation of insulin signaling reduces adiponectin, suppresses the activity of AMP-activated protein kinase (AMPK) in the liver, and impairs fatty acid oxidation, creating a lipid- and iron-rich environment for bacteria (Lin et al., 2013; Mohamed et al., 2016). However, although glycated hemoglobin reflects mean blood glucose levels over the preceding 8–12 weeks more stably and exhibits a linear association with the risk of invasiveness, its test is not routinely performed upon admission. In contrast, FBG is quickly accessible, inexpensive, and clinically feasible. Therefore, it is the preferred indicator for assessing uncontrolled blood glucose and predicting invasiveness in emergencies.

Smoking, alcohol consumption, and liver disease significantly increase the risk of IKPLAS. This is consistent with the predictive model in a study by Soochow University, which also identified liver disease as a key covariate for invasive infection in diabetic patients with KPLA (Feng et al., 2024). Nicotine in cigarettes inhibits the NF-κB pathway in alveolar macrophages, reducing IL-1β and TNF-α secretion and diminishing the initial clearance of Klebsiella pneumoniae (Chen et al., 2025). Harmful substances in smoke damage ciliated epithelial cells in the respiratory tract, impairing their ability to clear pathogens and leading to chronic bronchitis. This would compromise mucosal integrity, facilitating invasion of tissue by Klebsiella pneumoniae colonizing the respiratory tract (Zhang et al., 2021). Alcohol directly destroys intestinal tight junctions and elevates portal vein endotoxin levels. Consequently, hepatic Kupffer cells are activated to produce IL-10, creating an immunotolerant microenvironment (Chen et al., 2025). Acetaldehyde, a metabolite of ethanol, inhibits NADPH oxidase activity in neutrophils, reduces ROS-mediated bactericidal effects, and promotes synergistic immune evasion with the hypermucoviscous phenotype of KP (Mohamed et al., 2016). Therefore, smoking, alcohol consumption, and liver disease collectively amplify the invasive potential of KP through three pathways: mucosal barrier damage, immune deviation, and microenvironment hypoxia. Clinically, these three factors should be incorporated into the early warning scoring criteria for IKPLAS.

Elevated SOFA scores and septic shock are independent predictors for IKPLAS, which aligns with the conclusion of Sun et al.’s cohort study on multicenter community-acquired KP invasive infection conducted in Guangdong, China (Sun et al., 2025). Their study has demonstrated that each 1-point increase in SOFA score at admission is associated with an increase in mortality risk by 16%, with 61.9% of patients with KP invasive infection developing septic shock. The two studies collectively suggest that the key determinant of invasion and dissemination is multi-organ dysfunction reflected by SOFA, rather than the size of the hepatic lesion alone. Elevated SOFA scores are often accompanied by cytokine storms, that is, marked increases in IL-6 and TNF-α levels. This could upregulate ICAM-1 and EGFR in hepatocytes and bile duct epithelial cells, promoting adhesion and transcytosis of hypermucoviscous KP (Serban et al., 2021). Concurrently, the imbalance of coagulation and fibrinolysis (decreased platelet count and increased international normalized ratio) increases microthrombi in the hepatic sinusoid, leading to ischemia and necrosis of the abscess wall. This creates a “pressure valve” effect, allowing bacteria to enter the systemic circulation via the short hepatic vein-inferior vena cava pathway and induce shock. During shock, increased systemic vascular permeability further enhances the probability of distant migration (eyes, brain, lungs), forming a positive feedback loop. Although septic shock was identified as a significant risk factor, this finding was based on only 2 studies. The small sample size may lead to instability of effect estimates. Hence, the results should be interpreted with caution, and this risk factor needs to be further studied utilizing expanded literature. The cohort study in Hebei, China, though with a small sample size, has also demonstrated a strong association of high SOFA scores with gas formation in abscesses and decreased neutrophil ratios. This suggests that rapid gas production from necrotic liver parenchyma accelerates organ failure (Zhang et al., 2024). Therefore, in any patient with KPLA, a SOFA score ≥ 4 or early signs of shock should be regarded as a warning signal of progression to invasive KPLA. Particularly in patients with underlying conditions, proactive screening for hepatic lesions and intensified anti-infective therapy are essential to block this vicious cycle.

Phlebitis is the strongest and stablest independent predictor of IKPLAS, which strongly aligns with multicenter findings from studies of Gu et al., Li et al., and Wang et al (Wang et al., 2022; Li et al., 2023; Gu et al., 2025). Its mechanism transcends simple thrombotic occlusion: the hypermucoviscous KP activates TLR2/4 via magA/rmpA, elevating the expression of ICAM-1 and TF on vascular endothelium to cause *in situ* immunothrombosis (Lee et al., 2010). Bacterial capsular polysaccharides further shield the bacteria from neutrophil phagocytosis, enabling continuous release of bacterial emboli within the thrombus. These emboli disseminate along the portal-systemic-pulmonary axis or the hepatic vein-inferior vena cava-right heart-pulmonary circulation pathway, significantly increasing the rates of metastasis to the lungs, eyes, and CNS. The left hepatic lobe abscess is also frequently listed as a risk factor in multiple studies due to the anatomical characteristics of the lobe. The left hepatic vein branches are short with large diameters and share a common trunk with the middle and left hepatic veins directly connecting the inferior vena cava. Elevated abscess cavity pressure makes it easier for bacterial emboli to enter the systemic circulation. Besides, the left hepatic lobe and the left branch of the portal vein run in a straight line. This allows bacterial emboli in the portal vein to rapidly reach the splenic vein and superior mesenteric vein, inducing distant infections in the pancreas, spleen, and kidneys (Lee et al., 2005). Thus, phlebitis reflects the dissemination pathway formed by the interaction of the bacteria-vessel-immune triad, while left hepatic lobe abscess provides a geographical shortcut. The synergistic effect of both significantly increases the probability of IKPLAS.

Elevated levels of PCT, PT, and total bilirubin all increase the invasive potential of KPLA, which aligns with Sun et al.’s findings based on 291 cases of community-acquired infections (Sun et al., 2025). However, they only regard PCT as a marker of inflammation severity, without explaining why high PCT predicts distant dissemination. This can be explained by integrating the results of this meta-analysis with the mechanism study. When PCT > 10 ng/mL, the lactate concentration in the liver abscess microenvironment increases simultaneously, inhibiting cAMP via the PTS-CRP axis. This triggers high expression of capsular polysaccharide (CPS), enhancing bacterial resistance to phagocytosis and facilitating penetration of the hepatic sinusoids (Feng et al., 2023). Meanwhile, prolonged PT reflects activation of the KP endotoxin-TLR4 pathway, inducing extrinsic coagulation. Fibrin deposition forms bacterial microthrombi (Zhu et al., 2025), which shield bacteria from phagocytosis and embolize target organs such as the eyes and brain via the bloodstream. More importantly, total bilirubin > 20 μmol/L is often accompanied by compression of the bile duct or abscess rupture into the biliary tract. Decreased glycohyocholic acid concentration in the bile weakens its negative regulation of CPS synthesis, while conjugated bilirubin itself induces IL-1β release, amplifying the inflammatory cascade and providing an inflammatory soil for distant colonization of bacteria (Li et al., 2023). Thus, these three indicators are not merely passive outcomes of severe conditions but actively participate in the invasion process through the lactate-capsule axis, microthrombosis-dissemination axis, and bile-immune axis. Clinically, simultaneous elevation of PCT, PT, and total bilirubin should be regarded as a high-risk invasion window. In addition to adequate drainage and sensitive antimicrobial therapy, early management of potential migration sites and intervention in coagulation and cholestasis are expected to reduce distant infection rates.

The hypervirulent (HV) phenotype and K1 serotype are independent risk factors for IKPLAS, which aligns with Sun et al.’s findings from 291 cases of community-acquired infections in Guangdong, China (Sun et al., 2025). Lee et al. (2010) have further reported that HV-positive strains constitute 94.7% of IKPLAS strains, with 91.4% carrying rmpA/rmpA2. This suggests that rmpA activates the extracellular polysaccharide synthesis operon, enabling colonies to form > 10 mm mucous filaments to evade Kupffer cell phagocytosis in hepatic sinusoids and promote hematogenous dissemination. The K1 capsules inhibit complement C3 deposition and neutrophil chemotaxis because they contain magA-mediated 2,3-dihydro-2-deoxy-N-acetylmannuronic acid polymers, whose concentrations were positively associated with IL-6 level and SOFA score. Zhang et al. (2022) have identified significantly higher magA abundance in IKPLAS isolates with K1 serotype compared to non-invasive strains, accompanied by upregulation of iron transporter genes iucA and fepC. This indicates that K1 capsules and iron uptake synergistically enhance the tolerance to oxidative stress, which accelerates extrahepatic migration. Driven by advances in whole-genome and comparative genomics techniques, high-throughput sequencing has progressively revealed the virulence gene clusters, synthesis pathways of CPS, and essential regulatory elements associated with the HV phenotype. These findings establish a molecular basis for targeted intervention strategies (Liu et al., 2025). The HV phenotype and the K1 CPS serve as pivotal virulence markers characteristic of IKPLAS. The rmpA/rmpA2 genes activate the CPS cluster to synthesize a high-molecular-weight capsule (> 40 kDa) that impedes neutrophil phagocytosis. The magA gene facilitates intracellular transit, assisting in the bacterial translocation from the intestine to the liver via the portal vein. Strains of the K1 serotype utilize a dual iron transport system comprising aerobactin and iroBCDN, the expression of which is significantly increased in high-glucose conditions. This system captures host transferrin, leading to an NF-κB/IL-6 induced inflammatory cascade and the formation of multiple liquefied abscesses (Lee et al., 2009; Tu et al., 2024). The coexistence of diabetes, K1 serotype, and high levels of mucus elevates the risk of IKPLAS, indicating the co-selection of high virulence and host metabolism. This provides evidence to guide the precise control of infection and the development of targeted anti-virulence therapies.

It is noteworthy that abdominal symptoms (e.g., abdominal pain) are considered protective factors. When such symptoms are present, greater attention is paid to the risk of secondary infection during treatment, thereby preventing IKPLAS to some extent. Additionally, an elevated albumin level is also considered a protective factor. However, since this meta-analysis incorporated a limited number of studies, it is necessary to further validate the reliability of this conclusion.

This study may provide references for future treatment of patients with KPLA, assisting clinicians in preventing invasive infections. Clinicians should be watchful and implement preventive measures for patients with a history of smoking or alcohol consumption, especially those with diabetes, liver disease, elevated SOFA scores, septic shock, phlebitis, or left hepatic lobe abscesses. Precautions are also warranted for patients exhibiting abnormal laboratory indicators such as elevated PCT, FBG, PT, or total bilirubin, or those with isolates of the HV phenotype or K1serotype.

Among the variables involved in this study, marked heterogeneity is observed in 8 variables, including hypertension, SOFA, liver abscess size, simple leaf, septa, abnormal perfusion, glycated hemoglobin, and PLT. This diminishes the accuracy of pooled effect estimates. Additionally, 4 studies incorporated in this meta-analysis are rated medium-quality, which may also introduce potential bias and compromise the credibility of the results.

This study represents the first comprehensive meta-analysis of risk factors for IKPLAS through an extensive literature review. However, it has some limitations. First, a small number of studies are included. Some indicators are reported in only a few studies. This may affect the external validity and accuracy of the results. Second, publication bias is present. Gender-related publication bias is observed, reducing the reliability of results and necessitating further validation. Additionally, geographical limitations exist. The included studies are mainly from China and South Korea, suggesting that the identified risk factors may be more applicable to Asian populations. These limitations partially undermine the credibility of the conclusion of this study. Future studies should incorporate samples from more countries and regions to enhance the general applicability of identified risk factors. For instance, international multi-center studies covering Europe, North America, and South America should be carried out. Rigorously designed, large-scale prospective cohort studies are necessary to confirm the causal relationships between risk factors and invasive infections and to further validate the general applicability of the findings.

## 5 Conclusion

This study is the first comprehensive meta-analysis to identify key risk factors contributing to the progression of KPLA to IKPLAS: smoking, alcohol consumption, diabetes, underlying liver disease, elevated SOFA scores, septic shock, phlebitis, left hepatic lobe abscess, as well as elevated PCT, FBG, PT, or total bilirubin levels, and infection with strains of K1 serotype. Future large-scale prospective cohort studies should be conducted in expanded geographical regions to establish an evidence-based platform for precise early warning and intervention.
