# Supplementary material for: Risk factors for invasive Klebsiella pneumoniae liver abscess syndrome: a meta-analysis
Source: Front Cell Infect Microbiol. 2026 Jan 27;16:1749555. doi: 10.3389/fcimb.2026.1749555 (PMC12886464; doi:10.3389/fcimb.2026.1749555)

**Supplementary Materials**

**Supplementary Table S1.** Search strategy

**The search strategy (PubMed)**

| Search number | Query | Results |
| --- | --- | --- |
|  |  |  |
| 1 | Liver Abscess[MeSH Terms] | 8968 |
| 2 | "abscess of the liver"[Title/Abstract] OR "abscesses of the liver"[Title/Abstract] OR "hepatic abscess"[Title/Abstract] OR "hepatic abscesses"[Title/Abstract] OR "hepatic abscessus"[Title/Abstract] OR "liver abcess"[Title/Abstract] OR "liver abscess"[Title/Abstract] OR "liver abscesses"[Title/Abstract] OR "liver microabscess"[Title/Abstract] OR "liver microabscesses"[Title/Abstract] OR "microabscesses of the liver"[Title/Abstract] | 9638 |
| 3 | Klebsiella pneumoniae[MeSH Terms] | 20,045 |
| 4 | "b. friedlander"[Title/Abstract] OR "bacillus pneumoniae"[Title/Abstract] OR "Bacterium pneumoniae crouposae"[Title/Abstract] OR "bacterium pneumonie crouposae"[Title/Abstract] OR "friedlaender bacillus"[Title/Abstract] OR "friedlander bacillus"[Title/Abstract] OR "hyalococcus pneumoniae"[Title/Abstract] OR "k. pneumoniae"[Title/Abstract] OR "klebsiella crouposa"[Title/Abstract] OR "Klebsiella Pn"[Title/Abstract] OR "klebsiella pneumonia"[Title/Abstract] OR "Klebsiella pneumoniae"[Title/Abstract] OR "Klebsiella pneumoniae aerogenes"[Title/Abstract] OR "Klebsiella pneumoniae aerogenes"[Title/Abstract] OR "Klebsiella rhinoscleromatis"[Title/Abstract] OR "pneumobacillus"[Title/Abstract] | 36,906 |
| 5 | (#1 OR #2) AND (#3 OR #4) | 1,121 |

**The search strategy (embase)**

| Search number | Query | Results |
| --- | --- | --- |
| 1 | 'klebsiella pneumoniae'/exp | 72558 |
| 2 | 'b. friedlander':ab,ti,kw OR 'bacillus pneumoniae':ab,ti,kw OR 'bacterium pneumoniae crouposae':ab,ti,kw OR 'bacterium pneumonie crouposae':ab,ti,kw OR 'friedlaender bacillus':ab,ti,kw OR 'friedlander bacillus':ab,ti,kw OR 'hyalococcus pneumoniae':ab,ti,kw OR 'k. pneumoniae':ab,ti,kw OR 'klebsiella crouposa':ab,ti,kw OR 'klebsiella pn':ab,ti,kw OR 'klebsiella pneumonia':ab,ti,kw OR 'klebsiella pneumoniae':ab,ti,kw OR 'klebsiella pneumoniae aerogenes':ab,ti,kw OR 'klebsiella pneumoniae aerogenes':ab,ti,kw OR 'klebsiella rhinoscleromatis':ab,ti,kw OR 'pneumobacillus':ab,ti,kw | 51064 |
| 3 | 'liver abscess'/exp | 18335 |
| 4 | 'abscess of the liver':ab,ti,kw OR 'abscesses of the liver':ab,ti,kw OR 'hepatic abscess':ab,ti,kw OR 'hepatic abscesses':ab,ti,kw OR 'hepatic abscessus':ab,ti,kw OR 'liver abcess':ab,ti,kw OR 'liver abscess':ab,ti,kw OR 'liver abscesses':ab,ti,kw OR 'liver microabscess':ab,ti,kw OR 'liver microabscesses':ab,ti,kw OR 'microabscesses of the liver':ab,ti,kw | 13043 |
| 5 | (#1 OR #2) AND (#3 OR #4) | 1854 |

**The search strategy (Cochrane)**

| Search number | Query | Results |
| --- | --- | --- |
| 1 | MeSH descriptor: [Liver Abscess] explode all trees | 71 |
| 2 | ('abscess of the liver' OR 'abscesses of the liver' OR 'hepatic abscess' OR 'hepatic abscesses' OR 'hepatic abscessus' OR 'liver abcess' OR 'liver abscess' OR 'liver abscesses' OR 'liver microabscess' OR 'liver microabscesses' OR 'microabscesses of the liver' ):ab,ti,kw | 515 |
| 3 | MeSH descriptor: [Klebsiella pneumoniae] explode all trees | 141 |
| 4 | ('b. friedlander' OR 'bacillus pneumoniae' OR 'Bacterium pneumoniae crouposae' OR 'bacterium pneumonie crouposae' OR 'friedlaender bacillus' OR 'friedlander bacillus' OR 'hyalococcus pneumoniae' OR 'k. pneumoniae' OR 'klebsiella crouposa' OR 'Klebsiella Pn' OR 'klebsiella pneumonia' OR 'Klebsiella pneumoniae' OR 'Klebsiella pneumoniae aerogenes' OR 'Klebsiella pneumoniae aerogenes' OR 'Klebsiella rhinoscleromatis' OR 'pneumobacillus' ):ab,ti,kw | 929 |
| 5 | (#1 OR #2) AND (#3 OR #4) | 12 |

**The search strategy (WOS)**

| Search number | Query | Results |
| --- | --- | --- |
| 1 | TS=((abscess of the liver) OR (abscesses of the liver) OR (Hepatic Abscess) OR (hepatic abscesses) OR (hepatic abscessus) OR (liver abcess) OR (Liver Abscess) OR (Liver Abscesses) OR (liver microabscess) OR (liver microabscesses) OR (microabscesses of the liver)) and Preprint Citation Index (Exclude – Database) and Research Commons (Exclude – Database) | 25502 |
| 2 | TS=((b. friedlander) OR (Bacillus pneumoniae) OR (Bacterium pneumoniae crouposae) OR (bacterium pneumonie crouposae) OR (friedlaender bacillus) OR (friedlander bacillus) OR (Hyalococcus pneumoniae) OR (k. pneumoniae) OR (klebsiella crouposa) OR (Klebsiella Pn) OR (klebsiella pneumonia) OR (Klebsiella pneumoniae) OR (Klebsiella pneumoniae aerogenes) OR (Klebsiella pneumoniae aerogenes) OR (Klebsiella rhinoscleromatis) OR (pneumobacillus)) and Preprint Citation Index (Exclude – Database) and Research Commons (Exclude – Database) | 118212 |
| 3 | #2 AND #1 and Preprint Citation Index (Exclude – Database) and Research Commons (Exclude – Database) | 2340 |

**VIP**

| **序号** | **检索式** | **结果** |
| --- | --- | --- |
| **1** | **肝脓肿+肝脓疡** |  |
| **2** | **肺炎克雷伯菌** |  |
| **5** | **#1 AND #2** | **467** |

**万方**

| **序号** | **检索式** | **结果** |
| --- | --- | --- |
| **1** | **肝脓肿 OR 肝脓疡** |  |
| **2** | **肺炎克雷伯菌** |  |
| **5** | **#1 AND #2** | **825** |

**知网CNKI**

| **序号** | **检索式** | **结果** |
| --- | --- | --- |
| **1** | **肝脓肿 + 肝脓疡** |  |
| **2** | **肺炎克雷伯菌** |  |
| **5** | **#1 AND #2** | **353** |

**中国生物医学（sinomed）**

| **序号** | **检索式** | **结果** |
| --- | --- | --- |
| **1** | **肝脓肿 OR 肝脓疡** |  |
| **2** | **肺炎克雷伯菌** |  |
| **5** | **#1 AND #2** | **1465** |

**Supplementary Table S2 NOS Scoring Form**

**NEWCASTLE - OTTAWA QUALITY ASSESSMENT SCALE**

**CASE CONTROL STUDIES**

Note: A study can be awarded a maximum of one star for each numbered item within the Selection and Exposure categories. A maximum of two stars can be given for Comparability.

**Selection**

1) Is the case definition adequate?

a) yes, with independent validation ****

b) yes, eg record linkage or based on self reports

c) no description

2) Representativeness of the cases

a) consecutive or obviously representative series of cases ****

b) potential for selection biases or not stated

3) Selection of Controls

a) community controls ****

b) hospital controls

c) no description

4) Definition of Controls

a) no history of disease (endpoint) ****

b) no description of source

**Comparability**

1) Comparability of cases and controls on the basis of the design or analysis

a) study controls for _______________ (Select the most important factor.) ****

b) study controls for any additional factor **** (This criteria could be modified to indicate specific control for a second important factor.)

**Exposure**

1) Ascertainment of exposure

a) secure record (eg surgical records) ****

b) structured interview where blind to case/control status ****

c) interview not blinded to case/control status

d) written self report or medical record only

e) no description

2) Same method of ascertainment for cases and controls

a) yes ****

b) no

3) Non-Response rate

a) same rate for both groups ****

b) non respondents described

c) rate different and no designation

**NEWCASTLE - OTTAWA QUALITY ASSESSMENT SCALE**

**COHORT STUDIES**

Note: A study can be awarded a maximum of one star for each numbered item within the Selection and Outcome categories. A maximum of two stars can be given for Comparability

**Selection**

1) Representativeness of the exposed cohort

a) truly representative of the average _______________ (describe) in the community ****

b) somewhat representative of the average ______________ in the community ****

c) selected group of users eg nurses, volunteers

d) no description of the derivation of the cohort

2) Selection of the non exposed cohort

a) drawn from the same community as the exposed cohort ****

b) drawn from a different source

c) no description of the derivation of the non exposed cohort

3) Ascertainment of exposure

a) secure record (eg surgical records) ****

b) structured interview ****

c) written self report

d) no description

4) Demonstration that outcome of interest was not present at start of study

a) yes ****

b) no

**Comparability**

1) Comparability of cohorts on the basis of the design or analysis

a) study controls for _____________ (select the most important factor) ****

b) study controls for any additional factor **** (This criteria could be modified to indicate specific control for a second important factor.)

**Outcome**

1) Assessment of outcome

a) independent blind assessment ****

b) record linkage ****

c) self report

d) no description

2) Was follow-up long enough for outcomes to occur

a) yes (select an adequate follow up period for outcome of interest) ****

b) no

3) Adequacy of follow up of cohorts

a) complete follow up - all subjects accounted for ****

b) subjects lost to follow up unlikely to introduce bias - small number lost - > ____ % (select an adequate %) follow up, or description provided of those lost) ****

c) follow up rate < ____% (select an adequate %) and no description of those lost

d) no statement

**Supplementary Figure S1** Sensitivity analyses

ALT


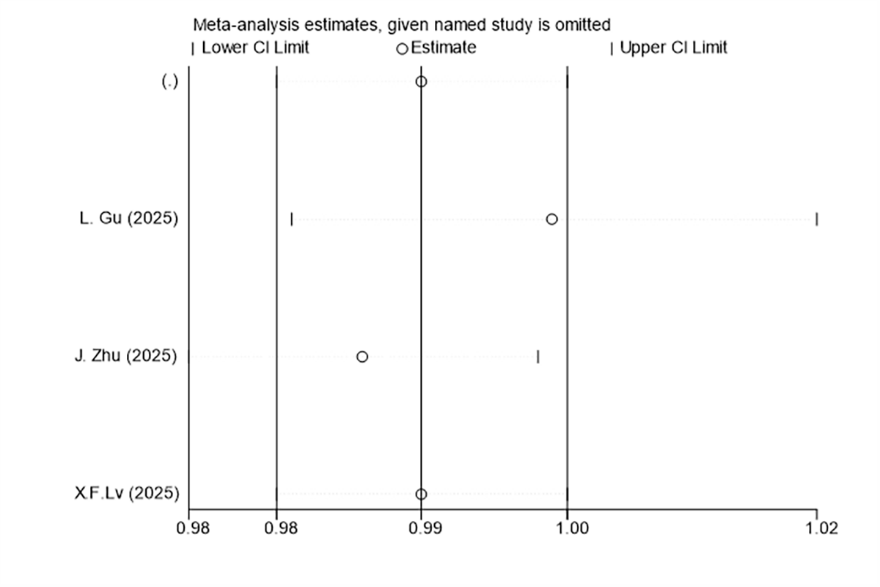


CRP


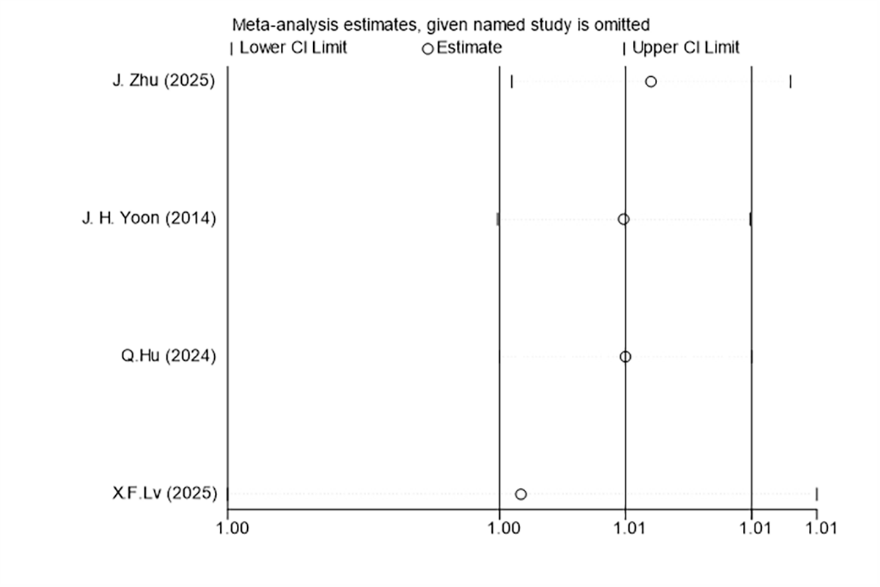


FBG


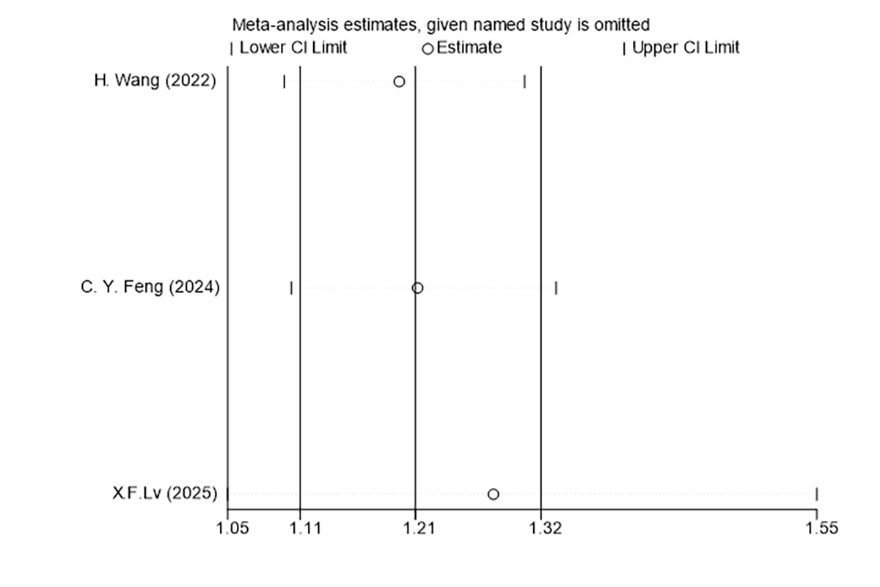


K1 serotype


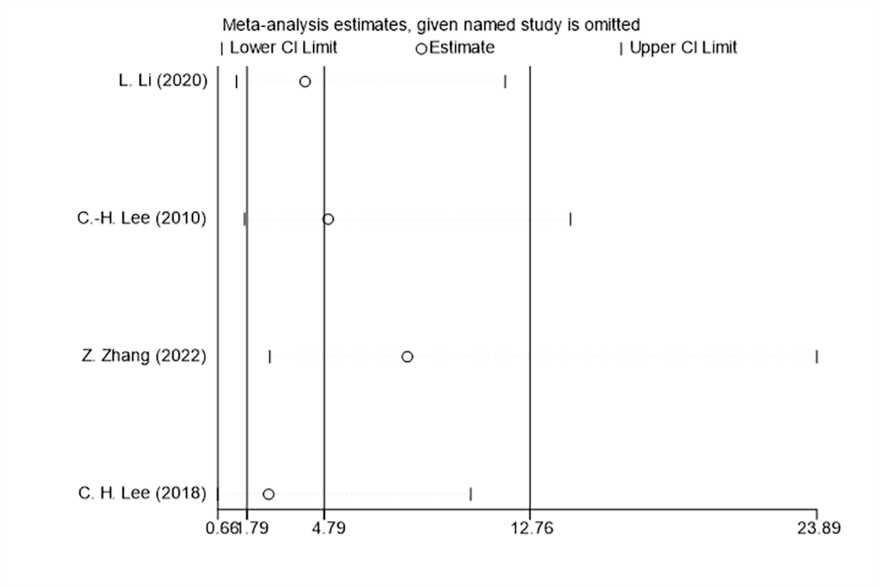


K2 serotype


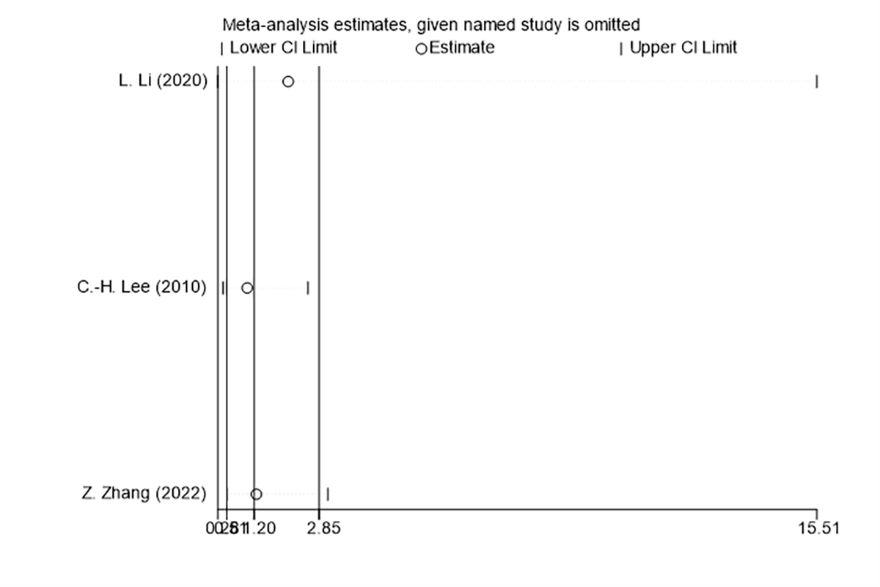


PCT


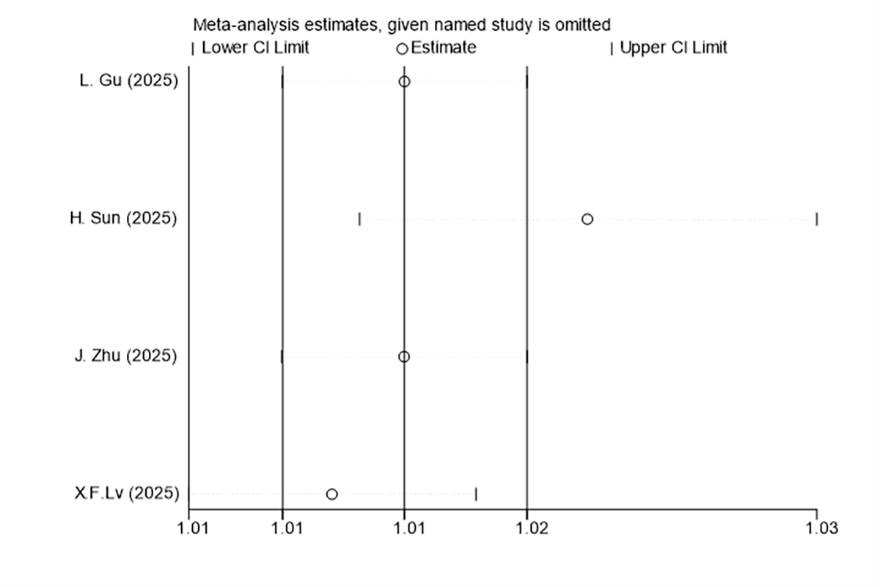


SOFA


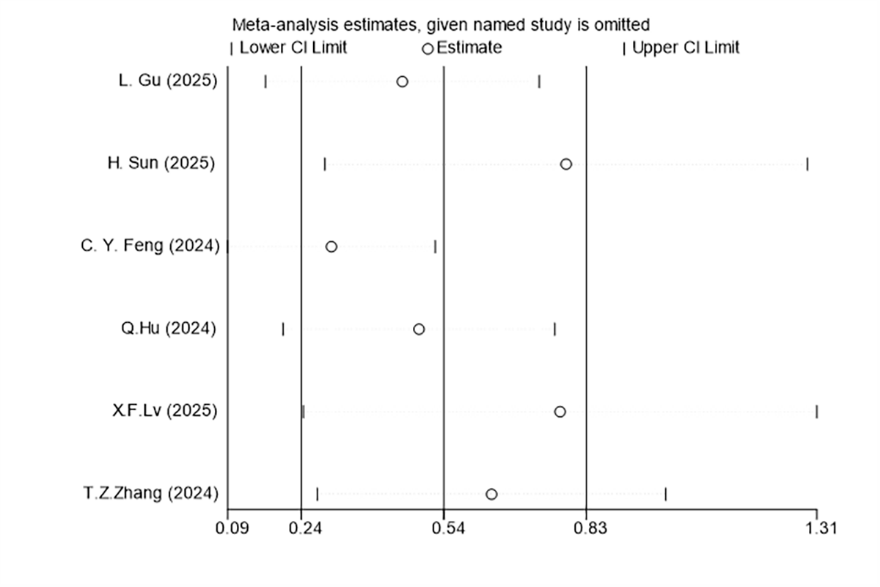


WBC


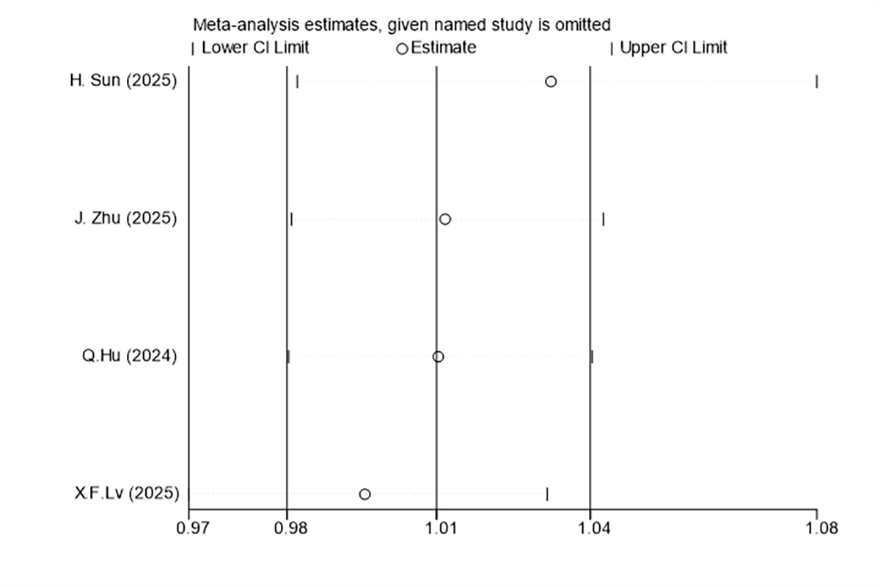


Single


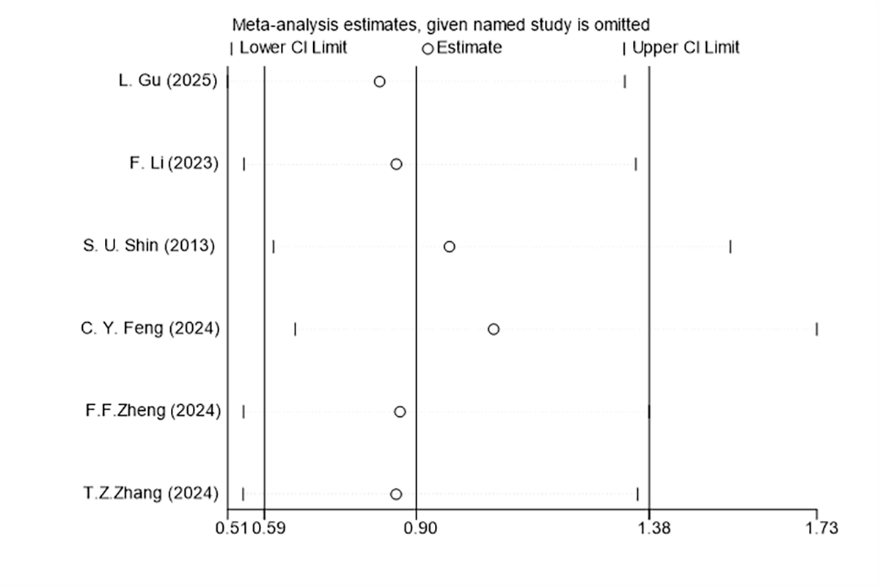


Biliary diseases


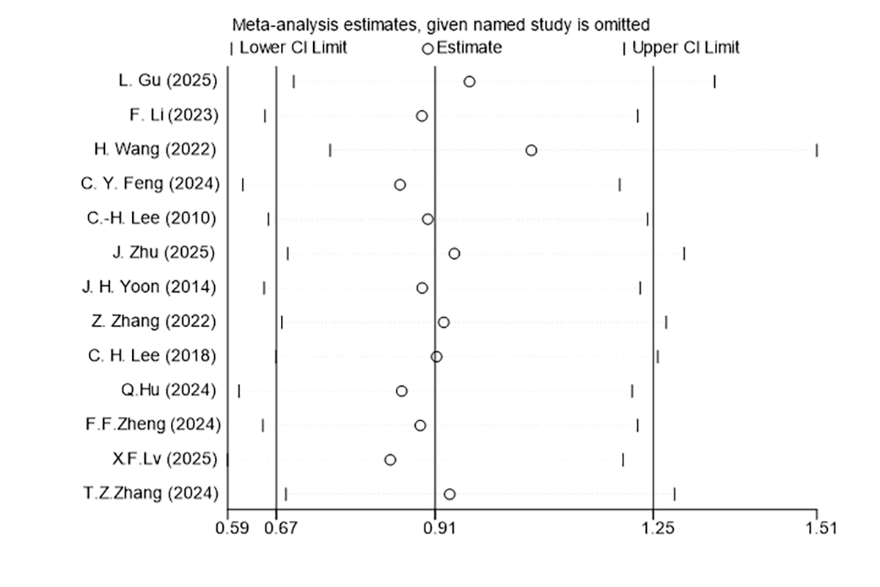


Multipl


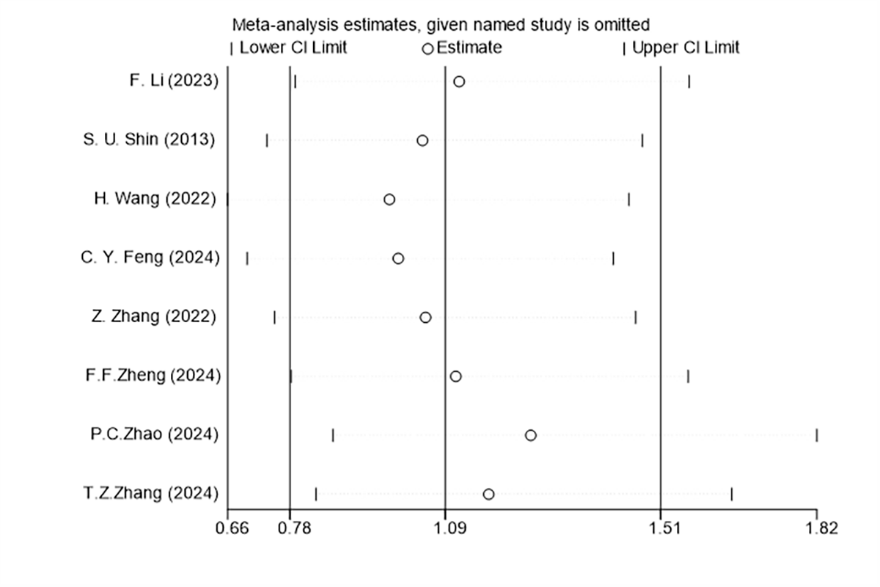


Fever


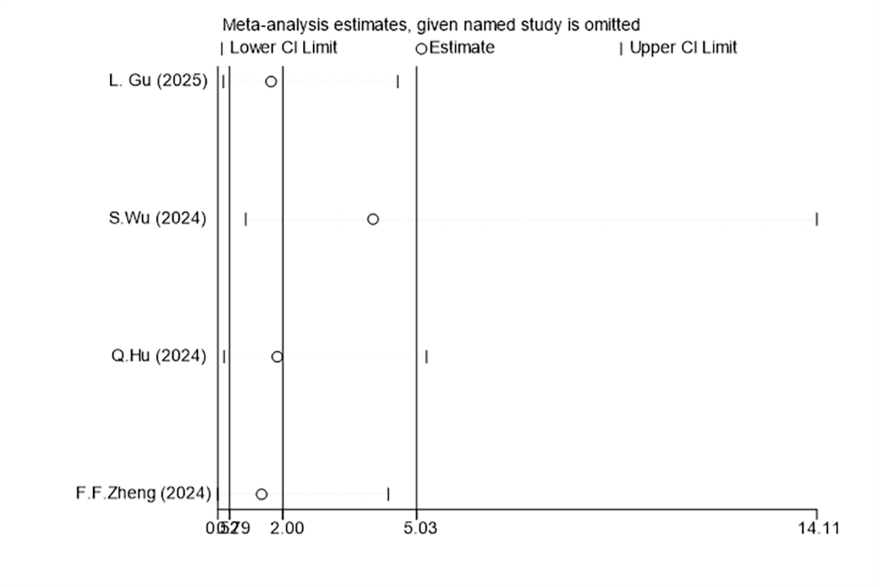


Abdominal surgical history


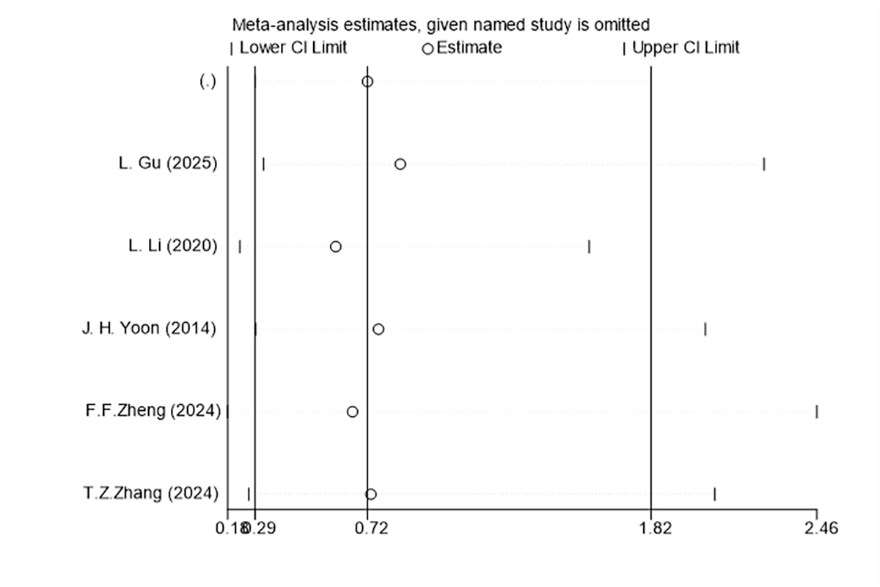


Abdominal symptoms


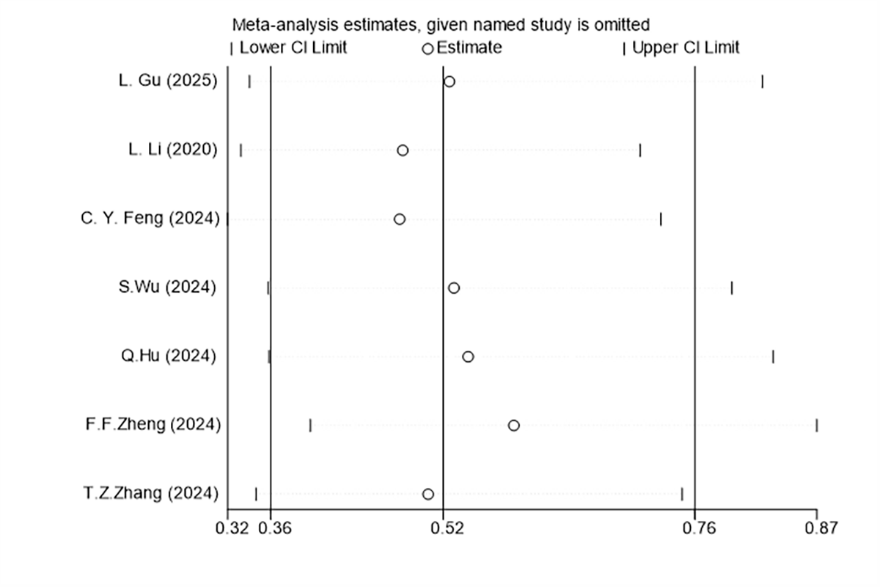


Liver disease


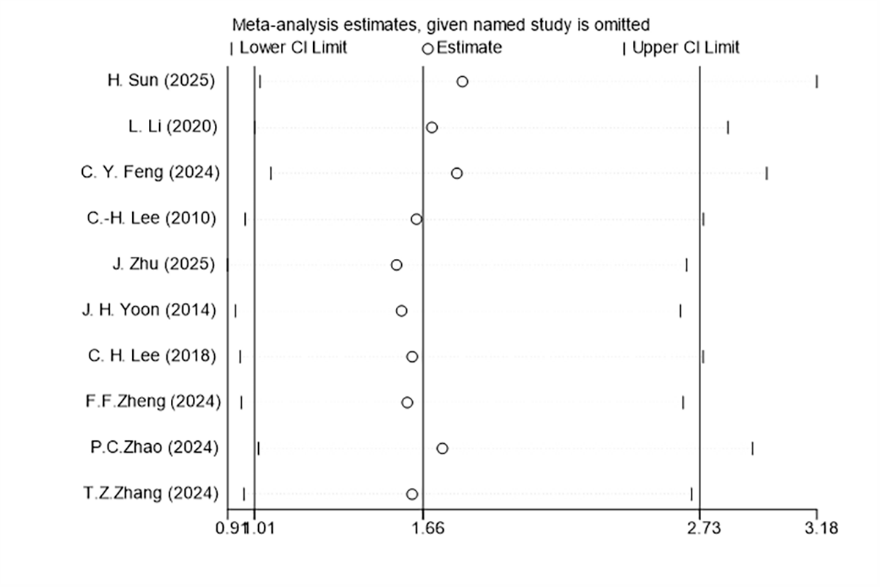


Abscess size


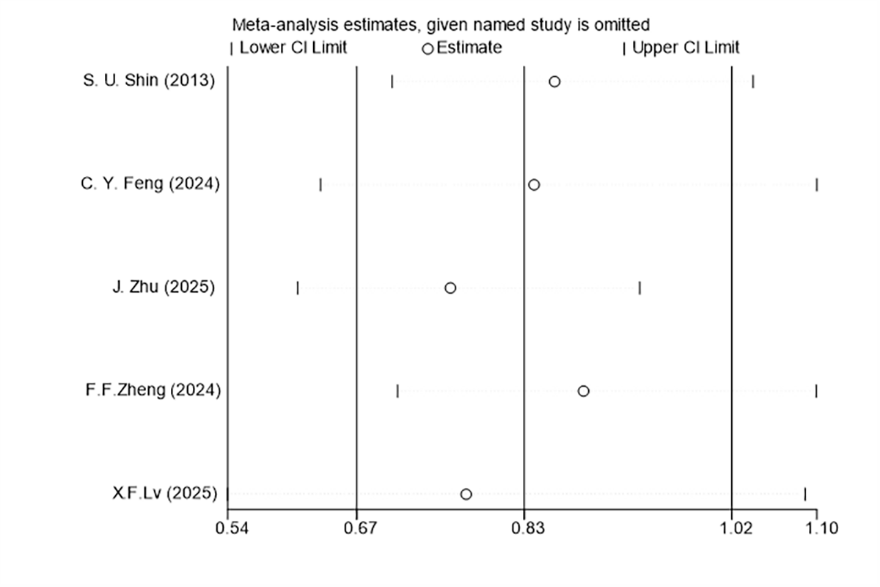


Right lobe


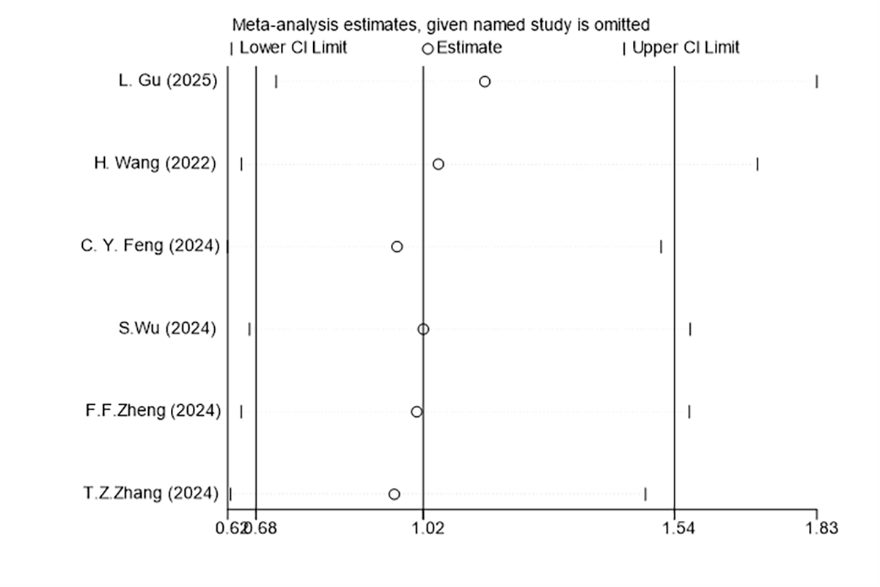


Left lobe


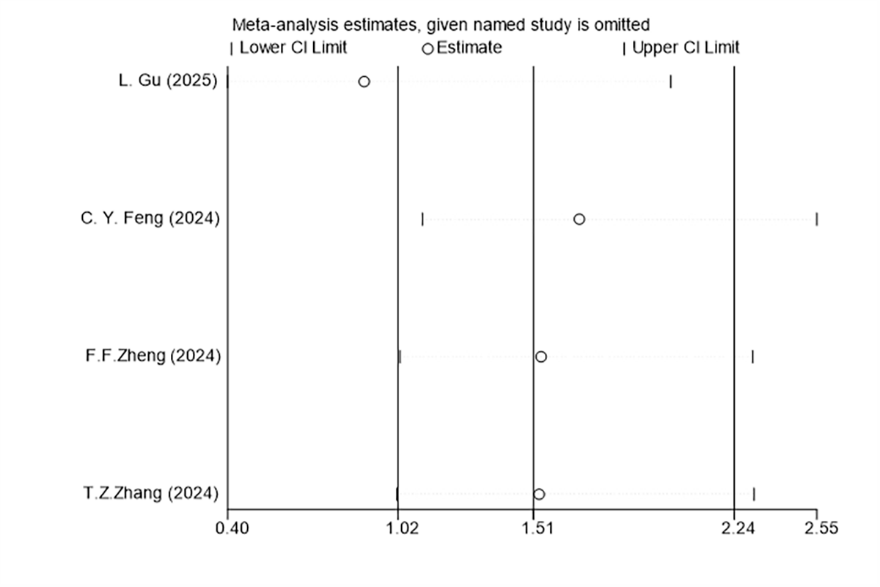


Hypertension


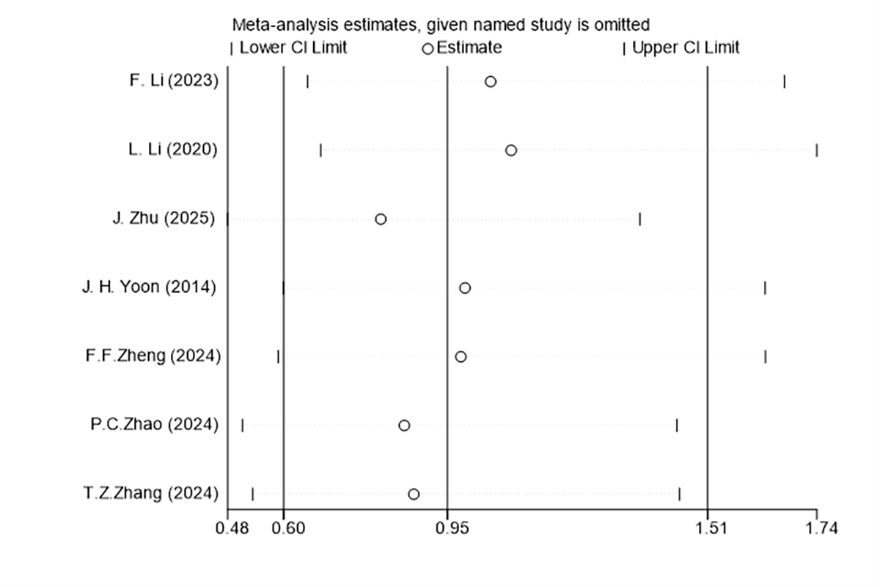


Hypervirulent phenotype


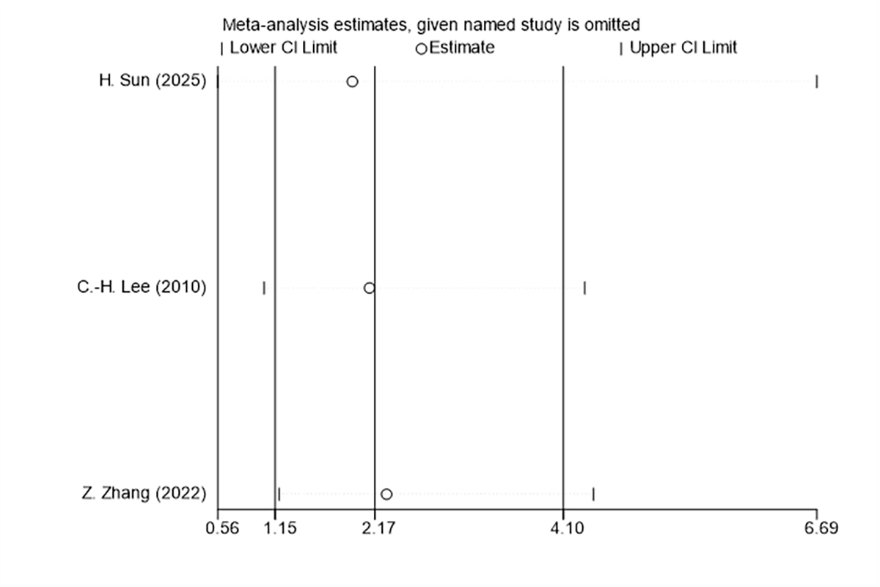


Septa


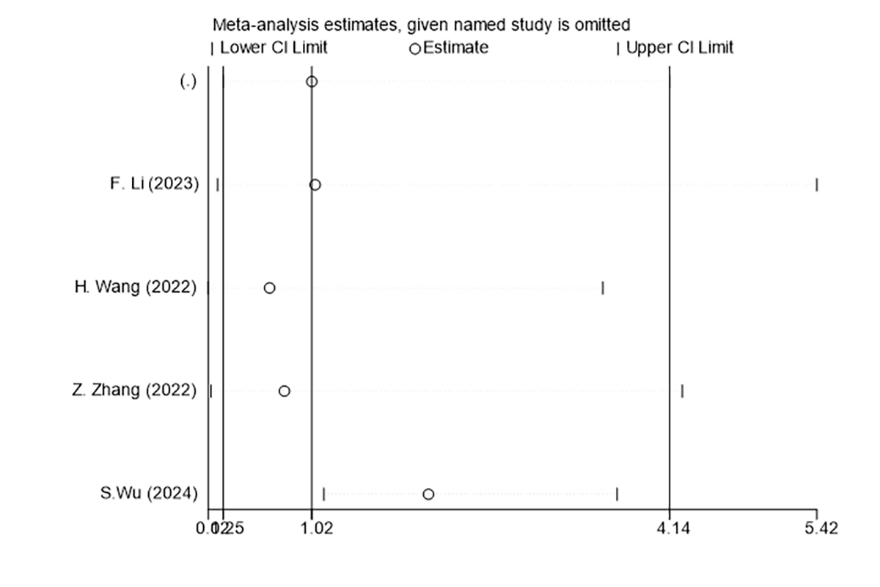


Chills/shivers


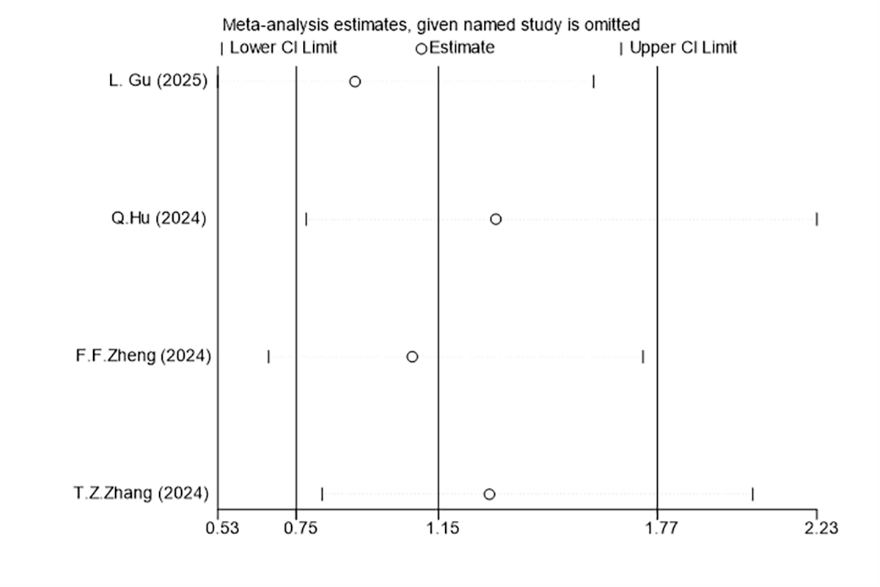


Phlebitis


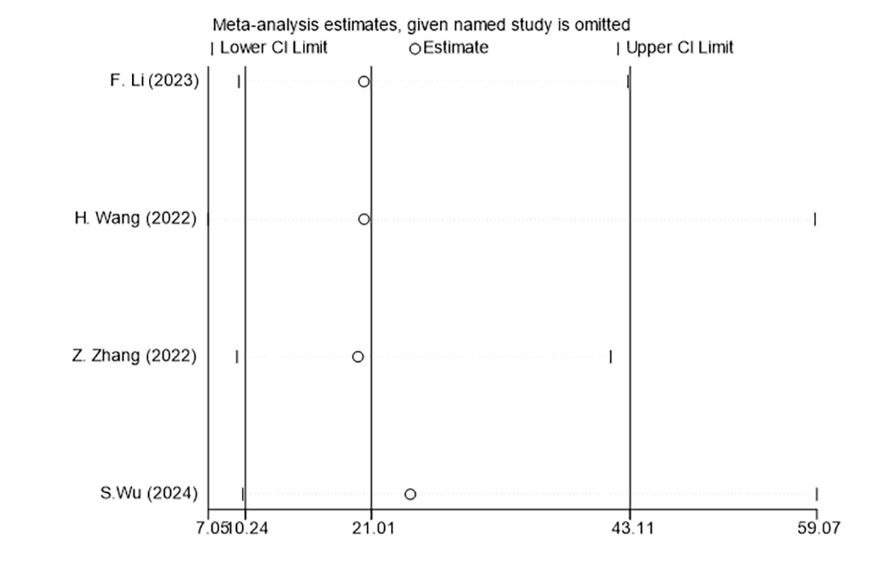


Chronic kidney disease


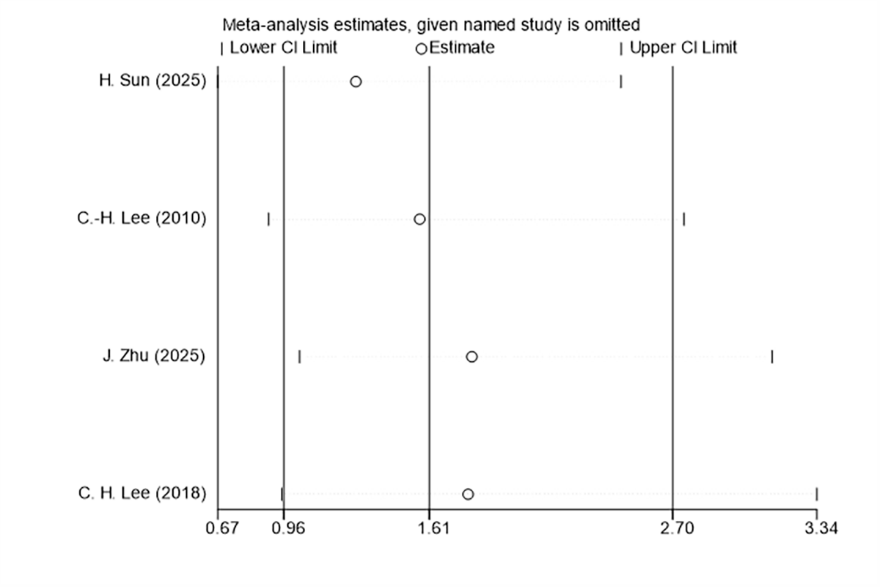


Chronic pulmonary disease


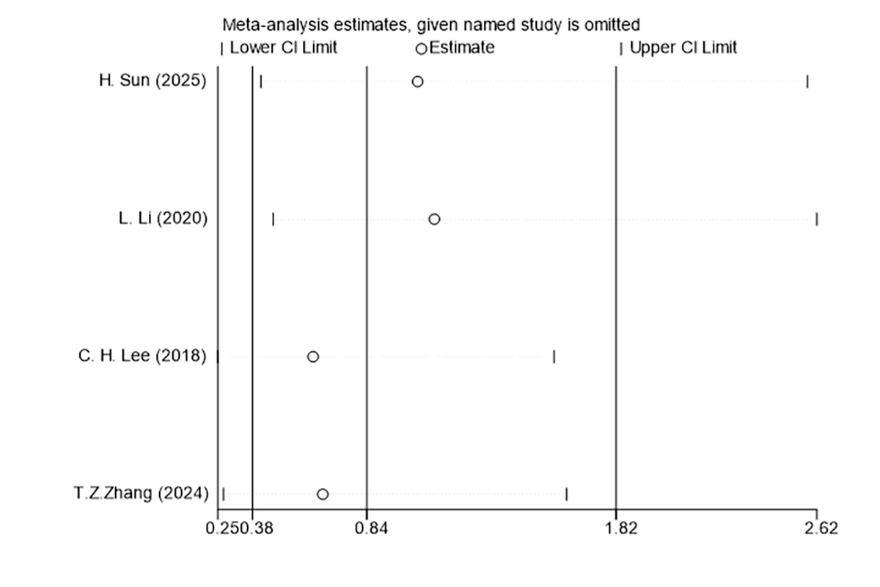


Drug-resistant strains


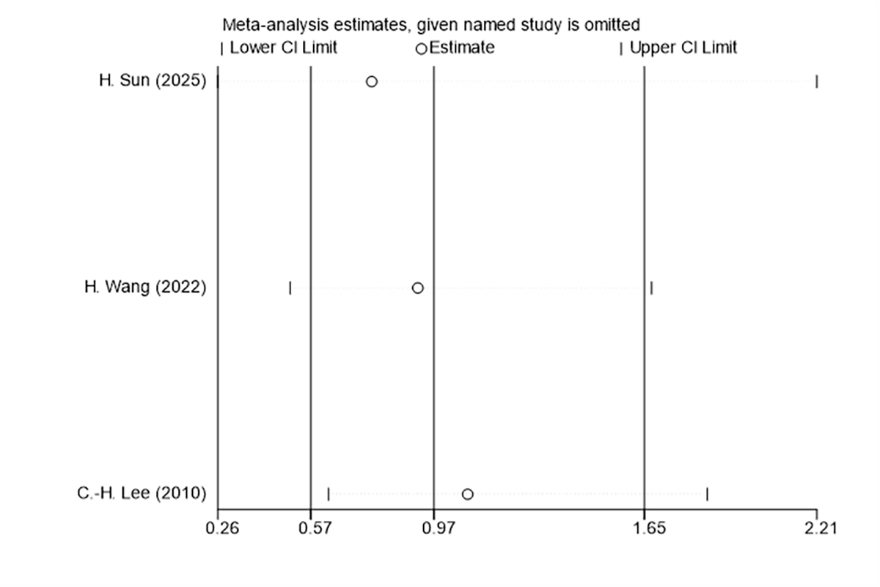


Age


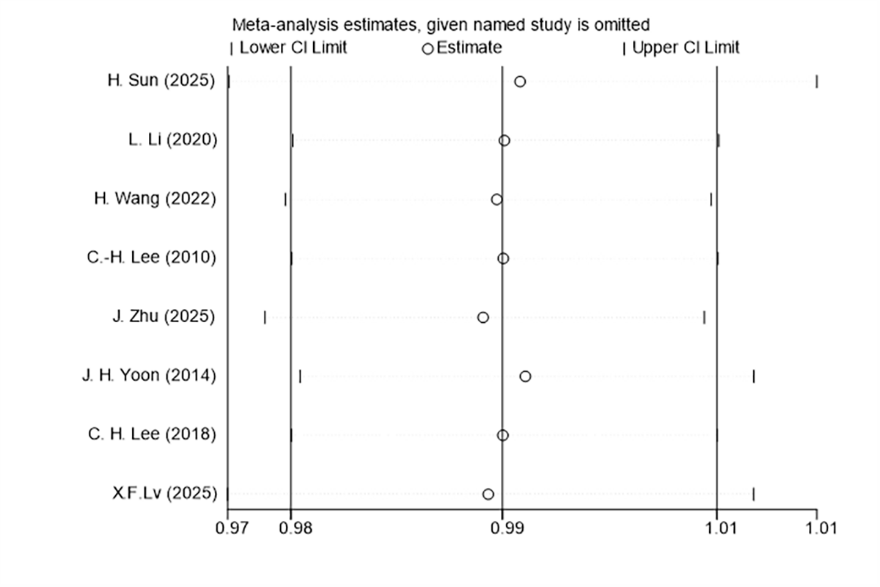


Gas in abscess


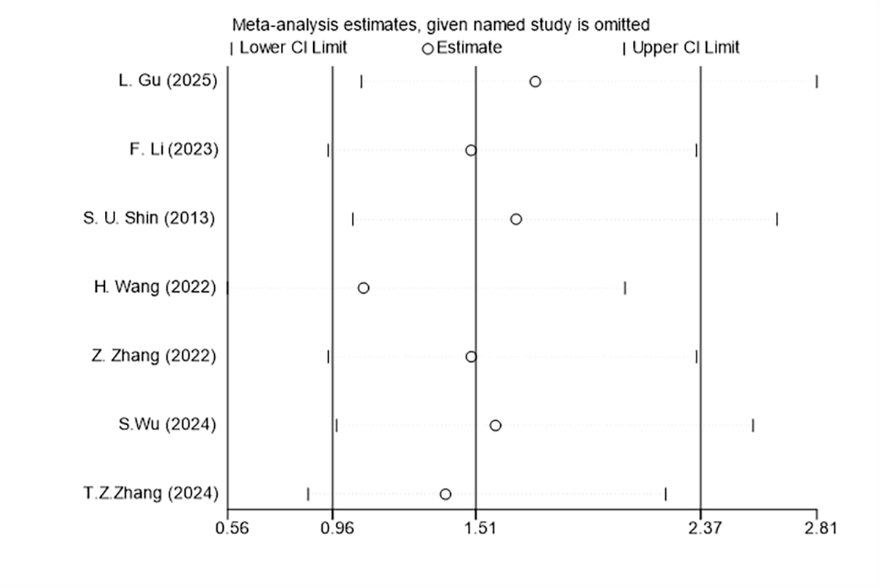


Neurologic disease


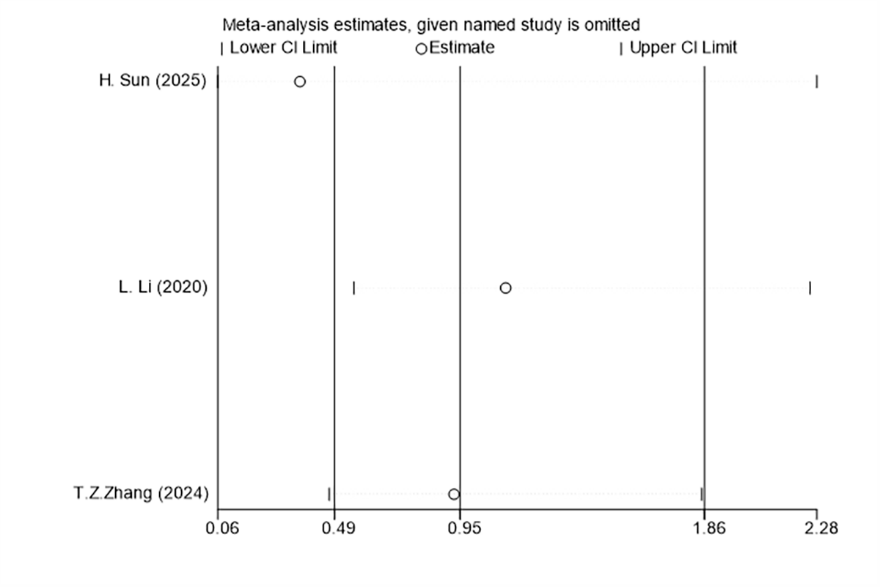


Poor appetite


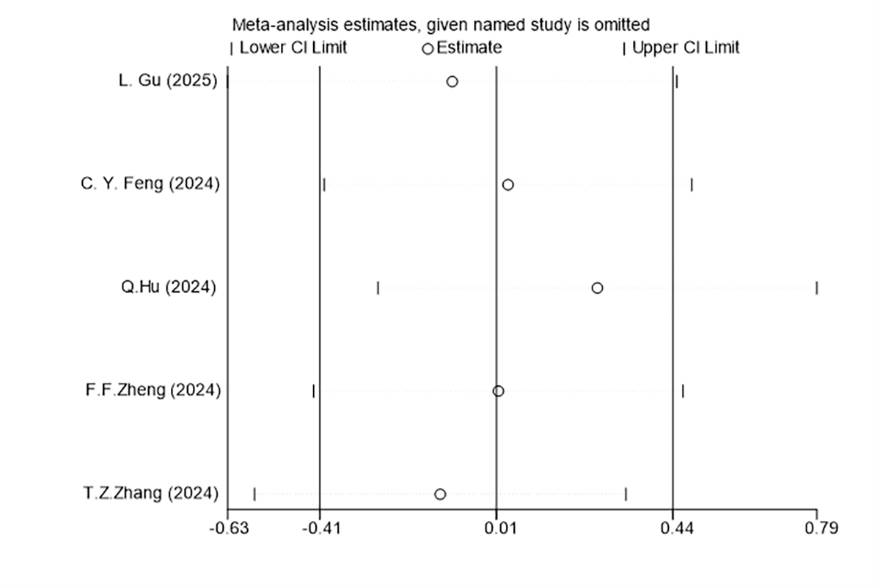


Glycated hemoglobin


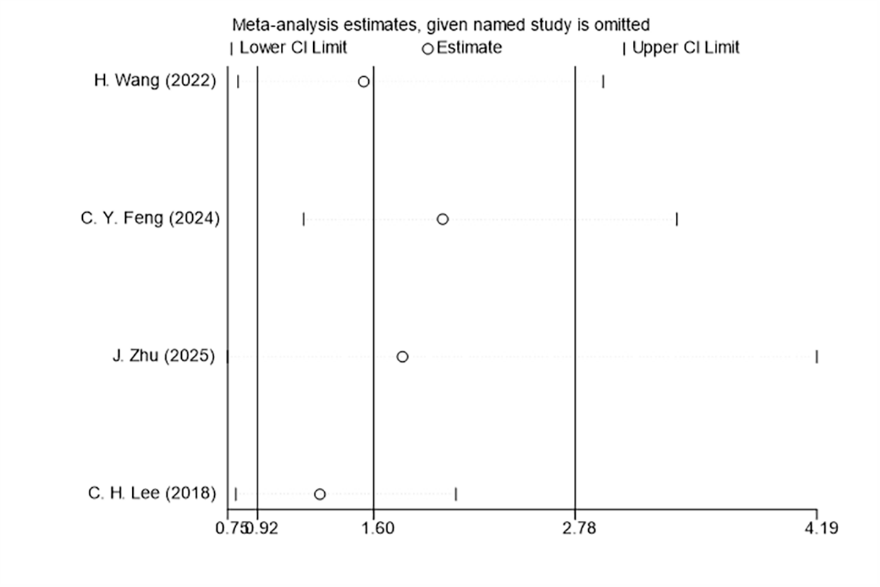


Diabetes mellitus


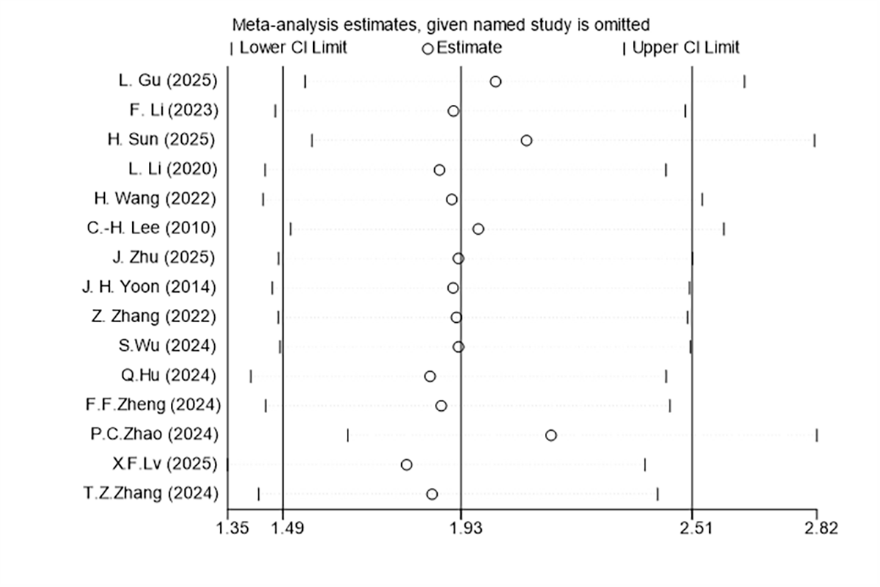


Smoking or drinking history


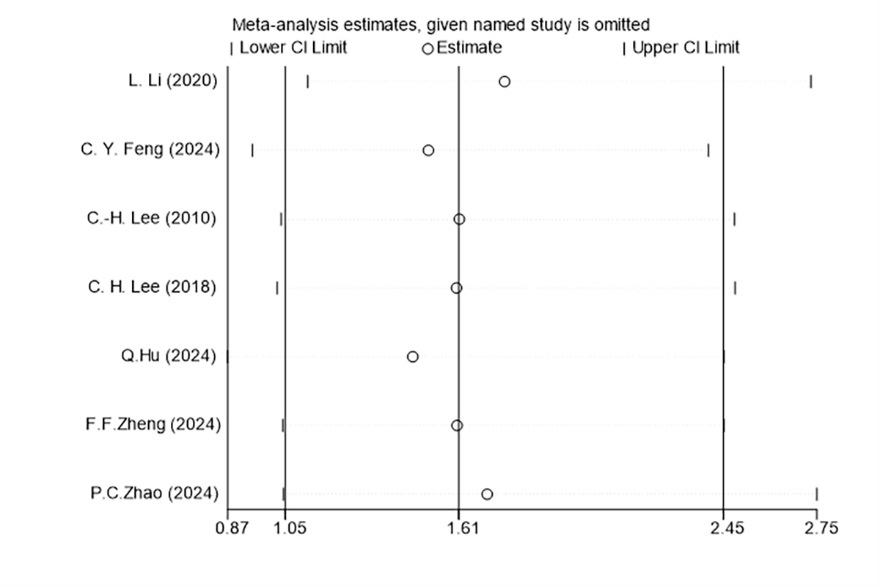


Cardiovascular disease


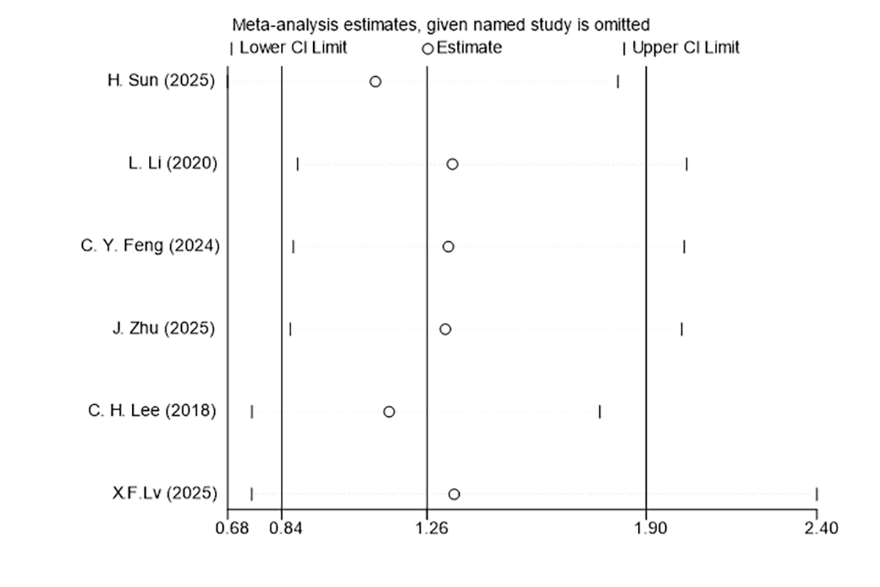


Gender (Male)


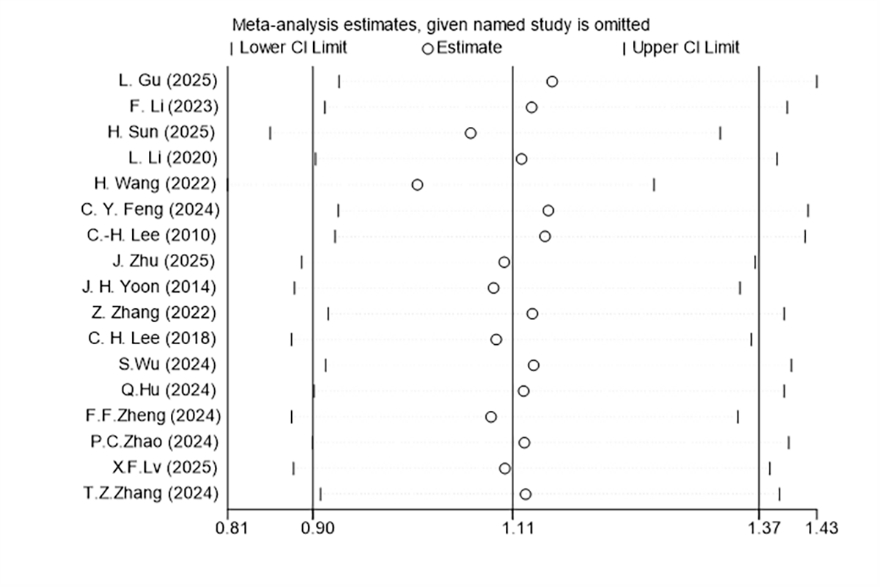


Weakness


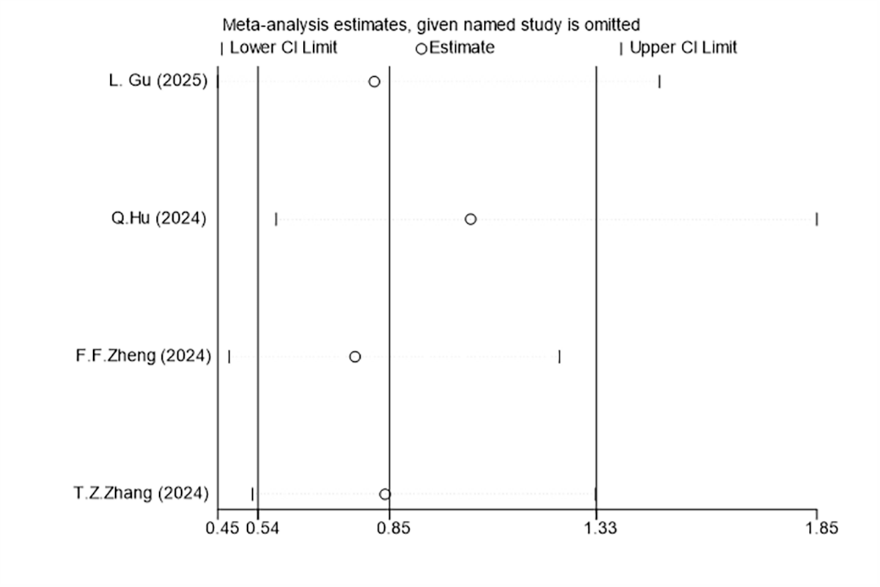


Hemoglobin


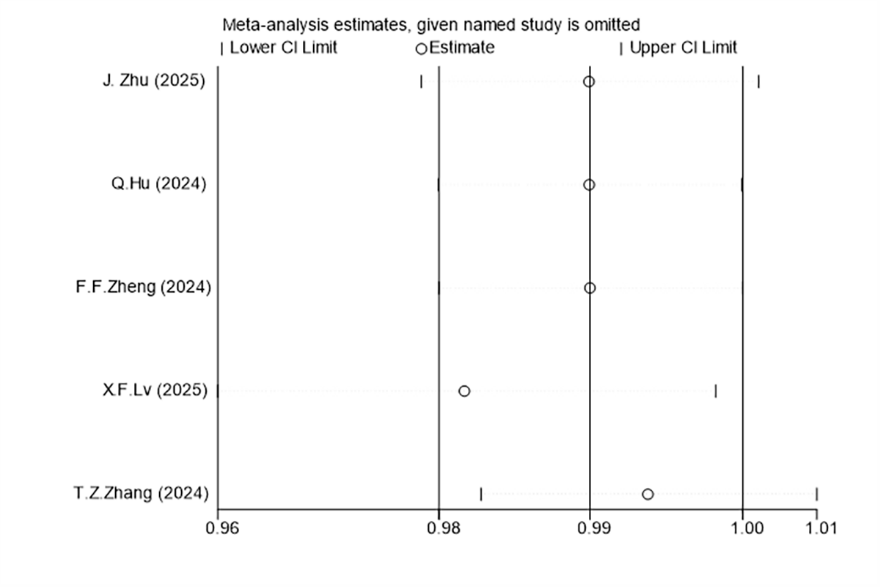


PLT


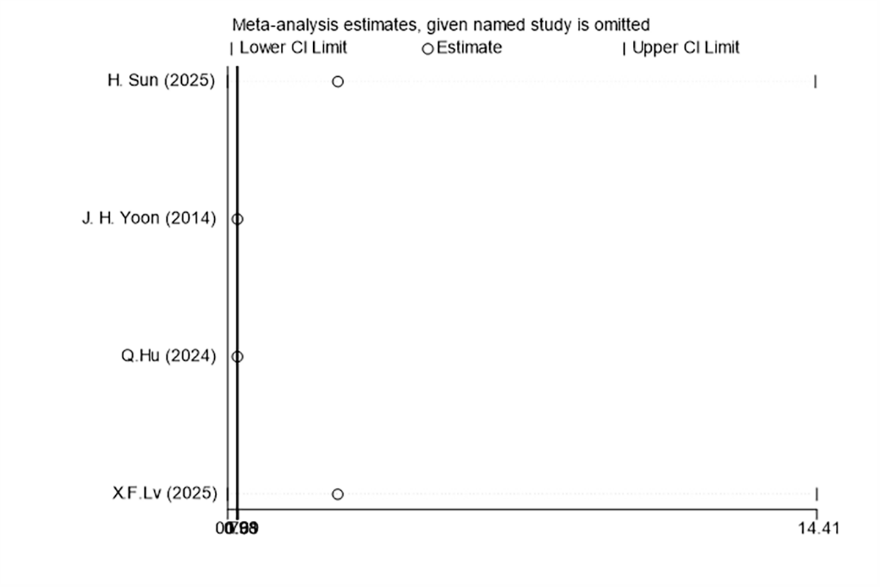


Neutrophile granulocyte


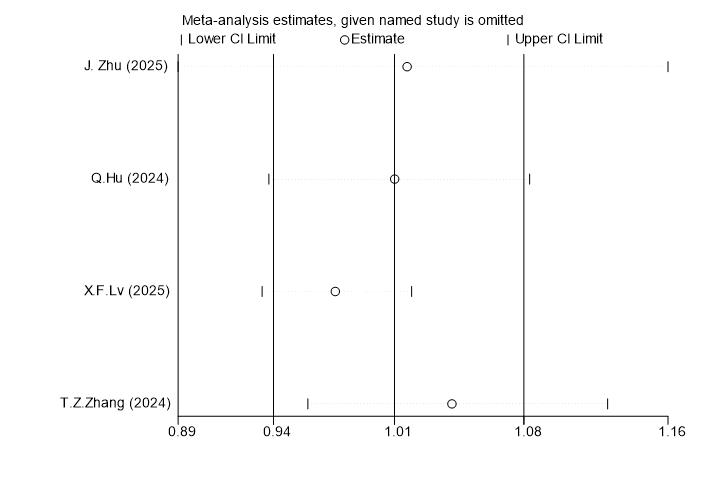


Tumor and autoimmune


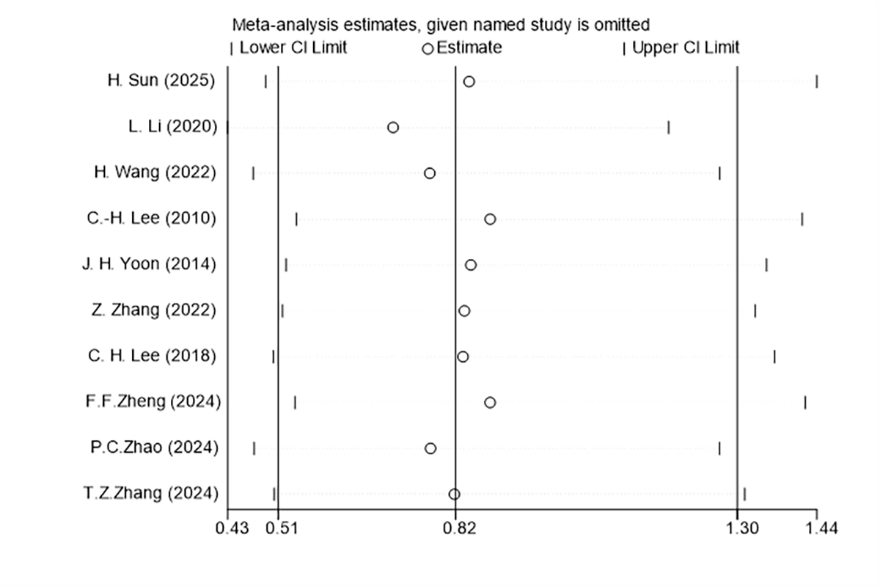


Both lobes


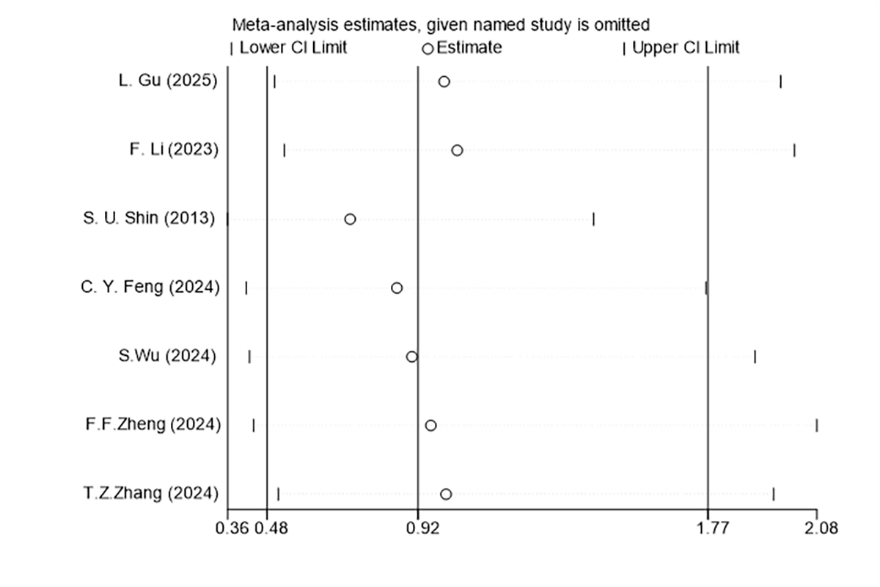

Supplement: Supplementary file 1 [file Table1.docx]
